# Family background and peer victimization are associated with generalized trust from adolescence to adulthood

**DOI:** 10.1038/s44271-026-00474-z

**Published:** 2026-05-22

**Authors:** David Bürgin, Laura Bechtiger, Clarissa Janousch, Annekatrin Steinhoff, Maria Picazo Mora, Denis Ribeaud, Manuel Eisner, Lilly Shanahan

**Affiliations:** 1 Jacobs Center for Productive Youth Development, University of Zurich, Zurich, Switzerland; 2 Child and Adolescent Psychiatric Research Department, University Psychiatric Hospitals, University of Basel, Basel, Switzerland; 3 Interdisciplinary Center for Psychopathology and Emotion Regulation, Department of Psychiatry, University Medical Center Groningen, Groningen, The Netherlands; 4 Department of Global Public Health, Karolinska Institute, Stockholm, Sweden; 5 Experimental and Clinical Pharmacopsychology, Department of Adult Psychiatry and Psychotherapy, University Hospital of Psychiatry Zurich, University of Zurich, Zurich, Switzerland; 6 University Hospital of Child and Adolescent Psychiatry and Psychotherapy, University of Bern, Bern, Switzerland; 7 Institute of Criminology, University of Cambridge, Cambridge, UK; 8 Department of Psychology, University of Zurich, Zurich, Switzerland

**Keywords:** Human behaviour, Development studies, Sociology

## Abstract

Trust in others plays an important role in psychological development. However, longitudinal and developmentally informed research on trust remains limited and fragmented across disciplines. This study aimed to investigate the developmental course of generalized trust from adolescence into young adulthood, and to test sociodemographic, family-level, and peer victimization-related antecedents of trust in an urban cohort. Generalized trust was self-reported at age 13, 15, 17, 20, and 24 in the Zurich Project on Social Development from Childhood to Adulthood (z-proso; *n* = 1,481). Mean-level and rank-order stability of trust were described. Growth trajectories were modeled using longitudinal linear mixed models (LMMs). Sociodemographic and childhood family-level factors, and longitudinal peer victimization were tested as predictors of trust. Mean-levels of trust followed a curvilinear trajectory characterized by an early decline from age 13 to 17 (Cohen’s d = -.48) and a slight increase from age 17 to 24 (d = .11). Rank-order stability of trust increased throughout adolescence (Spearman’s r = .40 to 0.58). Notably, socioeconomic disadvantage and migration background were each associated with widening trust disparities over time. Individuals from more disadvantaged backgrounds showed lower and more slowly recovering trust trajectories compared to their more advantaged peers. Maternal trust and parental involvement were associated with higher trust, whereas aversive parenting and parental divorce or separation were linked with lower trust. Peer victimization was associated with lower trust, while increases in peer victimization showed the strongest associations with trust at ages 17 and 20. Generalized trust is a malleable yet increasingly stable construct from adolescence into adulthood. Childhood and adolescent risk and protective factors are important contributors to trust development and should be considered when designing developmentally informed policies and interventions.

## Abstract (web summary)

Using a longitudinal cohort, this study models generalized trust from ages 13–24 and test sociodemographic, family, and peer-level predictors. Trust declines in midadolescence, partly recovers, and shows widening inequalities linked to disadvantaged backgrounds and peer victimization.

## Introduction

Generalized trust (i.e., trust in most others) is fundamental not only for individual psychological development and well-being, but also for social capital and societal functioning^1–3^. Conversely, distrust is associated with heightened threat perception and a sense of unpredictability, both of which have been identified as risk mechanisms underlying various mental and physical health conditions^4–7^. Although early socialization experiences in childhood and adolescence lay the foundation for trust development^8,9^, trust is rarely examined from a developmental-longitudinal perspective. Characterizing the trajectory of generalized trust from adolescence into young adulthood is important, as it indicates when trust is most malleable, how it changes, and when social disparities emerge or widen. This study aims to investigate the developmental course of trust from adolescence to young adulthood, along with its predictors, using data from an urban cohort study with trust measurement spanning 11 years.

### Child development and trust

Trust constitutes an important foundation for healthy psychological development and long-term well-being^3,8^. It underlies and results from the formation of secure bonds with caregivers and peers, which are essential for developing core capacities such as self-regulation and socioemotional competence^10,11^. Within resilience research, trust is recognized as a key factor that promotes adaptation by facilitating close relationships, exploration, and autonomy^12,13^. More broadly, trust likely influences young people’s access to and benefit from socio-ecological resources, including supporting institutions, systems, and professionals^14–16^. Thus, understanding the developmental antecedents of trust is essential for promoting children’s adaptation, well-being, and positive engagement within society.

### Conceptualizing generalized trust

Interpersonal trust is commonly categorized into behavioral, particularized, and generalized trust^17–19^. Behavioral trust refers to situational and reciprocal trust behavior typically observed in experimental settings, whereas particularized trust refers to trust directed toward known individuals or specific groups. In contrast, generalized trust reflects an individual’s tendency to trust most people, including strangers^17–20^, and is also referred to as propensity to trust, dispositional trust, trustfulness, or trust beliefs^17,18,21^.

The heritability of generalized trust is relatively low: meta-analytic estimates suggest a heritability of approximately 27.4%^22^, indicating that environmental influences play a substantial role in its development^23^. Cultural perspectives propose that trust is shaped early in life through intergenerational transmission and cultural socialization, resulting in a relatively stable disposition. In contrast, experiential perspectives emphasize the plasticity of trust and the continuing influence of environmental factors throughout the lifespan^24,25^.

### The developmental course of generalized trust

Adolescence and young adulthood are formative periods of behavioral and emotional development, marked by major social transitions and the assumption of new roles and responsibilities^26,27^. Examining trust during adolescence can clarify how environmental factors shape its development during this formative period. Although there is a growing empirical literature on trust in childhood and adolescence, including studies using extensive questionnaires and behavioral trust games, this work is dispersed across disciplines and often uses diverse labels for related constructs. As a result, and because much of the existing research is cross-sectional or based on short-term longitudinal designs, it is difficult to integrate these findings into a coherent, longer-term picture of generalized trust development. While behavioral trust has more frequently been studied longitudinally, e.g.,^20,28,29^, longitudinal research of generalized trust over time is rare, with few exceptions. Two accelerated longitudinal studies, each with two repeated measurements across multiple age cohorts, have identified non-linear developmental trajectories and increasing rank-order stability of trust throughout adolescence^30–33^. Characterizing the developmental course and stability of generalized trust trajectories from adolescence into young adulthood is important, as it indicates when trust is most malleable and whether changes are linear or non-linear. This information on cohort trajectories helps situate risk and protective factors within a developmental context and highlights sensitive periods when interventions or policy changes may shape young people’s trust in others, and when social disparities in trust may emerge.

### Predictors of generalized trust

Research consistently demonstrates that trust is shaped by multiple contextual levels, aligning closely with Bronfenbrenner’s socioecological model of development and contemporary developmental models of multisystemic resilience^34–37^. *Sociodemographic factors* are important, as they systematically influence both access to opportunities that foster trust and exposure to risks that erode it^1,37,38^. Specific sociodemographic predictors of lower generalized trust include low socioeconomic status (SES), low educational attainment, and belonging to a migrant or racially minoritized group^32,38–40^. Additionally, adult women tend to report slightly lower generalized trust than men^38^, though this difference has not consistently been replicated in adolescent samples^33,41^. *Family-level factors* influence the development of trust through mechanisms such as fostering emotional safety, modeling social norms, and shaping cognitive schemas regarding the trustworthiness of others^30,33,42^. Parental behaviors and family structure are particularly influential, as they define a child’s primary developmental context. Parents’ own levels of distrust and caution towards unfamiliar others, as well as family disruptions, such as parental divorce or separation, or separation from one’s parents, have been associated with lower trust^33,42–45^. Furthermore, harsh parenting and parental violence have been linked to lower levels of trust^38,46^. In contrast, positive parent-child relationship quality and parenting styles characterized by compassion and democratic values are associated with higher and increasing generalized trust^33,38^. During adolescence, peer-level factors become increasingly salient^26,47^. Peer victimization during childhood and early adolescence has consistently been associated with lower trust^32,48–50^, whereas supportive and positive peer experiences have been shown to foster increases in trust^30^.

### Toward a more comprehensive understanding of trust development

Understanding how generalized trust develops from adolescence into young adulthood requires examining the full range of sociodemographic, family-level, and peer-related factors that shape it. While previous research has highlighted important predictors of trust in adolescence, it has often been limited in scope, examining a narrow set of predictors, relying on single-timepoint trust measurements, or modeling linear declines between just two time points. Studies are needed that test a broader set of predictors of non-linear trust trajectories across adolescence and into young adulthood within the same individuals. Such approaches are well-suited to capturing the complexity of trust development and to examining whether different predictors exert varying influences across different ages.

### The current study

This study investigated the developmental course of generalized trust from adolescence to young adulthood, using data from an ongoing urban cohort study. Specifically, we examined sociodemographic, childhood family-level, and adolescent peer-level predictors of generalized trust development across five measurement timepoints over an 11-year follow-up period. First, we investigated the developmental course and stability of generalized trust from age 13 to age 24, examining both mean-level and rank-order stability. Second, we tested sociodemographic predictors of trust trajectories, including sex, migration background, family SES, and educational attainment. We also tested whether these associations differed by age. Third, we examined the associations of family-level factors assessed during childhood and early adolescence, including maternal trust, parental involvement, parental divorce or separation, and aversive parenting, with generalized trust trajectories, testing whether these associations varied by age and adjusting for sociodemographic factors. Fourth, we analyzed how within- and between-person variations in peer victimization throughout adolescence were associated with generalized trust, again testing for age-varying associations and adjusting for sociodemographic and family-level factors. Peer victimization was included as a longitudinal predictor as it was measured at every time point and reflects the increasing developmental salience of peer relationships during adolescence and young adulthood^51^.

## Methods

### Study design and participants

Data came from the ongoing longitudinal Zurich Project on Social Development from Childhood to Adulthood (z-proso^52^). In 2004, 1675 children from 56 primary schools were selected as the target sample from 90 randomly selected public schools in Zurich, Switzerland’s largest city, using a cluster-stratified randomized sampling approach. The sample was broadly representative of first graders attending public schools in Zurich. The last data collection occurred in 2022 at age 24. Adolescents provided written informed consent for their participation, while parents could opt out on behalf of their children up to age 16. Data collection was typically conducted in groups of 5 to 25 participants, using paper-and-pencil questionnaires in classroom settings up to age 17, and computer-administered surveys in a computer lab at ages 20 and 24 (5–15% completing surveys online). The surveys typically took about 60 minutes. Participants received cash compensation ranging from roughly $30 at age 13 to $150 at age 24. Further details on study procedures are reported elsewhere^52–54^.

We investigated trust trajectories of *n* = 1481 participants longitudinally, with data collected at ages 13, 15, 17, 20, and 24. 48.3% (*n* = 715) of participants sex-assigned at birth collected from school records were female, 51.7% (*n* = 766) of participants sex-assigned at birth were male. Half of the sample (49.6%) had both parents born abroad (representing over 80 countries of origin), whereas 90% of participants themselves were born in Switzerland. The highest average household International Socio-Economic Index of Occupational Status (ISEI-index) in adolescence was 45.7 (SD = 19.2). By age 24, participants’ highest achieved educational levels were as follows: 8.9% below apprenticeship, 36.9% completed apprenticeship, 15.2% vocational baccalaureate, 13.3% baccalaureate, 11.8% vocational tertiary degree, and 13.9% academic tertiary degree.

For the present analyses, the analytic sample comprised all cohort members who participated in at least one of the five focal waves (ages 13, 15, 17, 20, or 24) and who provided at least one valid generalized trust score, yielding an analytic sample of *n* = 1,481 adolescents and young adults. The data used in this study are covered by two ethics approvals for the z-proso study: Phase 5/Wave 8 (2017–2020; approval number: 2018.2.12) and Phase 6/Wave 9 (2021–2024; approval number: 21.12.13), both from the Ethics Committee of the Faculty of Arts and Social Sciences, University of Zurich. In accordance with Swiss Law, participants provided informed consent at all measurement timepoints (ages 13, 15, 17, 20, and 24). At ages 13 and 15, parental passive consent was obtained (parents could refuse participation of their children). Parental data from earlier waves did not need ethical approval in accordance with Swiss law. Parents, however, in each of these waves, provided written informed consent.

### Measures

#### Outcomes

*Generalized trust* is conceptualized as adolescents’ and young adults’ propensity to trust unfamiliar others to be reliable, trustworthy, and fair^55,56^. At ages 13, 15, 17, 20, and 24, participants responded to three items adapted from the World Values Survey Questionnaire^57^: “Most people can be trusted,” “People usually try to help other people,” and “Most people try to be fair.” Responses were recorded on a four-point Likert scale ranging from 1 = fully untrue to 4 = fully true^54,58^. Internal consistency was moderate to high across waves (Cronbach’s alpha: age 13 = 0.74; age 15 = 0.78; age 17 = 0.83; age 20 = 0.84; age 24 = 0.86). For descriptive purposes, we used a mean score and for modeling purposes, a standardized mean trust score as an indicator for generalized trust.

#### Predictors

##### Sociodemographic predictors

Sex assigned at birth was obtained from school records at age 7 and dummy coded (0 = male; 1 = female). Migration background was assessed dichotomously and dummy coded (0 = at least one parent born in Switzerland; 1 = both parents were born abroad)^54^. Family SES was assessed using the ISEI (Ganzeboom, De Graaf^59^), with the highest family ISEI recorded score between ages 11 and 15 used in the analyses^54^. Participants’ highest educational attainment at age 24 was self-reported and coded into six levels: below apprenticeship, apprenticeship, vocational baccalaureate, baccalaureate, vocational tertiary degree, academic tertiary degree^54^.

#### Family-level predictors

##### Family-level predictors were assessed at ages 8 and 13

*Maternal generalized trust* was assessed from study participants’ mothers at child age 8 using three binary items adapted from the World Value Survey ( ~ 5% of parent interviews were conducted with fathers or other caregivers)^60^. Higher values on the resulting total trust score (0–3) indicated greater maternal trust^55,56^. *Parental involvement* at age 13 was assessed via child reports using six items from the Alabama Parenting Questionnaire (APQ), capturing positive aspects of parent–child engagement^61^. *Parental separation or divorce* was reported by the participants at age 13. *Aversive parenting* was measured using five APQ items that assessed physical and harsh verbal discipline by parents at age 13^61^. Detailed information on all these measures is provided in the Supplementary Material Table 1 including internal consistency measures.

#### Peer-level predictors

 Peer victimization was assessed at the ages 13, 15, 17, 20, and 24 using five items of the Zurich Bullying Scale^52,62^, capturing experiences of victimization in the past 12 months. Items asked if participants were ‘purposely ignored or excluded’, ‘mocked, or insulted’, ‘hit, bitten or kicked or hair-pulled’, ‘sexually harassed’, or if others ‘took or destroyed belongings. Detailed information on this measure is provided in the Supplementary Material Table 1 including internal consistency measures per study wave.

### Data analyses

We included *n* = 1481 participants who completed at least one valid measure of generalized trust at ages 13, 15, 17, 20, or 24. Sociodemographic characteristics were reported using descriptive statistics, and a Pearson correlation matrix of all study variables is provided in Supplementary Fig. 1. Missing data in predictor variables were addressed using multivariate imputation by chained equation (MICE), with *k* = 40 imputations, implemented in the ‘mice’-package. Further details on the imputation process are provided in Supplementary Methods 1.

To address our first aim, we examined the mean-level and rank-order stability of generalized trust across five timepoints (ages 13, 15, 17, 20, and 24). Descriptive statistics, Spearman rank correlations, and semiparametric paired Wilcoxon tests were conducted on complete cases to assess age-related differences and stability. Additionally, we modeled growth curves of generalized trust using longitudinal linear mixed models (LMMs). For that, we used maximum likelihood estimation to facilitate model comparisons and the “bobyqa” optimizer (up to 100,000 iterations) to ensure robust model convergence^63^. Age was coded as years since the baseline assessment, with possible values of 0, 2, 4, 7, and 11^64^. Multiple baseline models were compared, varying in structure and specifications of fixed and random effects for age and age². The best-fitting model included fixed linear and quadratic age terms, a random slope for the Level‑1 age predictor, and a person-level random intercept (Level 2; see Supplementary Methods 2, Supplementary Tables 2–3).

Because participants were originally sampled within schools, we examined school-level clustering in generalized trust using empty random-intercept models with school (at cohort entry) as the grouping variable. Intraclass correlations were very small in adolescence (ICC = 0.003–0.016) and modest in young adulthood (ICC = 0.046–0.061), indicating that only a small share of variance was attributable to between-school differences. Given these low ICCs and the dissolution of the original school clusters after the transition to upper secondary education, we used more parsimonious two-level models (age within individuals) with random intercepts and slopes at the person level rather than adding a school level.

To examine sociodemographic and family-level time-invariant predictors of generalized trust on the person-level (level 2 of the model; aims 2 and 3), we extended the baseline models by including additional sociodemographic and family-level variables as fixed effects. We estimated a series of hierarchical LMMs:

1. a model including sociodemographic predictors as fixed effects,
2. a model including sociodemographic predictors and their interactions with age and age² as fixed effects,
3. a model including family-level predictors as fixed effects, adjusting for sociodemographic covariates, and
4. a model including both fixed effects and interaction terms between family-level predictors and age/age², while adjusting for sociodemographics (see Supplementary Table 4).

To further explore SES-related effects, we fitted two exploratory, descriptive models that replaced family SES with participants’ highest educational attainment at age 24 and its interaction with age as correlates of generalized trust trajectories (see Supplementary Table 5). These models were used solely to describe how trajectories differ by later educational pathways and were not interpreted causally.

To address our fourth aim, we extended previous models by including time-variant peer victimization as a predictor of generalized trust. To distinguish between-person from within-person effects of peer victimization, we calculated each participant’s mean level of peer victimization across all waves (between-person effect, level 2) and the wave-specific deviation from this mean (within-person effect, level 1). The within-person component (age-specific deviation) was included both as a fixed effect and as a random slope, capturing individual differences in how fluctuations in victimization relate to changes in generalized trust over time. We then fitted two hierarchical LMMs (full model specifications and results are presented in Supplementary Table 6):

1. a model including both between-person and within-person peer victimization as fixed effects, adjusting for sociodemographic and family-level covariates, with within-person peer victimization modeled as a random slope, and
2. a model including these fixed effects along with their interactions with age and age².

Estimates from models in imputed datasets were pooled using Rubin’s rule to account for both within- and between-imputation variability^65^. As no common method for the pooling of random effects exists, we used random effects from single models run on merged datasets. Model assumptions for LMMs (e.g., linearity, normality of residuals, homoscedasticity, outliers and influential data points) were checked for all models run on these merged datasets using the *check_models* function from the performance package^66^. All models fulfilled model requirements.

The statistical software used was R through RStudio (Version 4.2.2; 2022-10-31; Boston, MA, USA)^67^. All continous variables were standardized prior to inclusion in all models, and categorical variables were dummy coded. Analyses were not preregistered prior to conducting the analyses. All statistical tests were two-sided, with significance levels defined as p < 0.05. Full model equations and annotated analysis code are provided in an online repository (see: 10.17605/OSF.IO/PC9HB). All data used in this study are available from the z-proso project via SWISSUbase under restricted access conditions^68,69^.

## Results

### Developmental course of generalized trust from adolescence to young adulthood

**Fig. 1:**
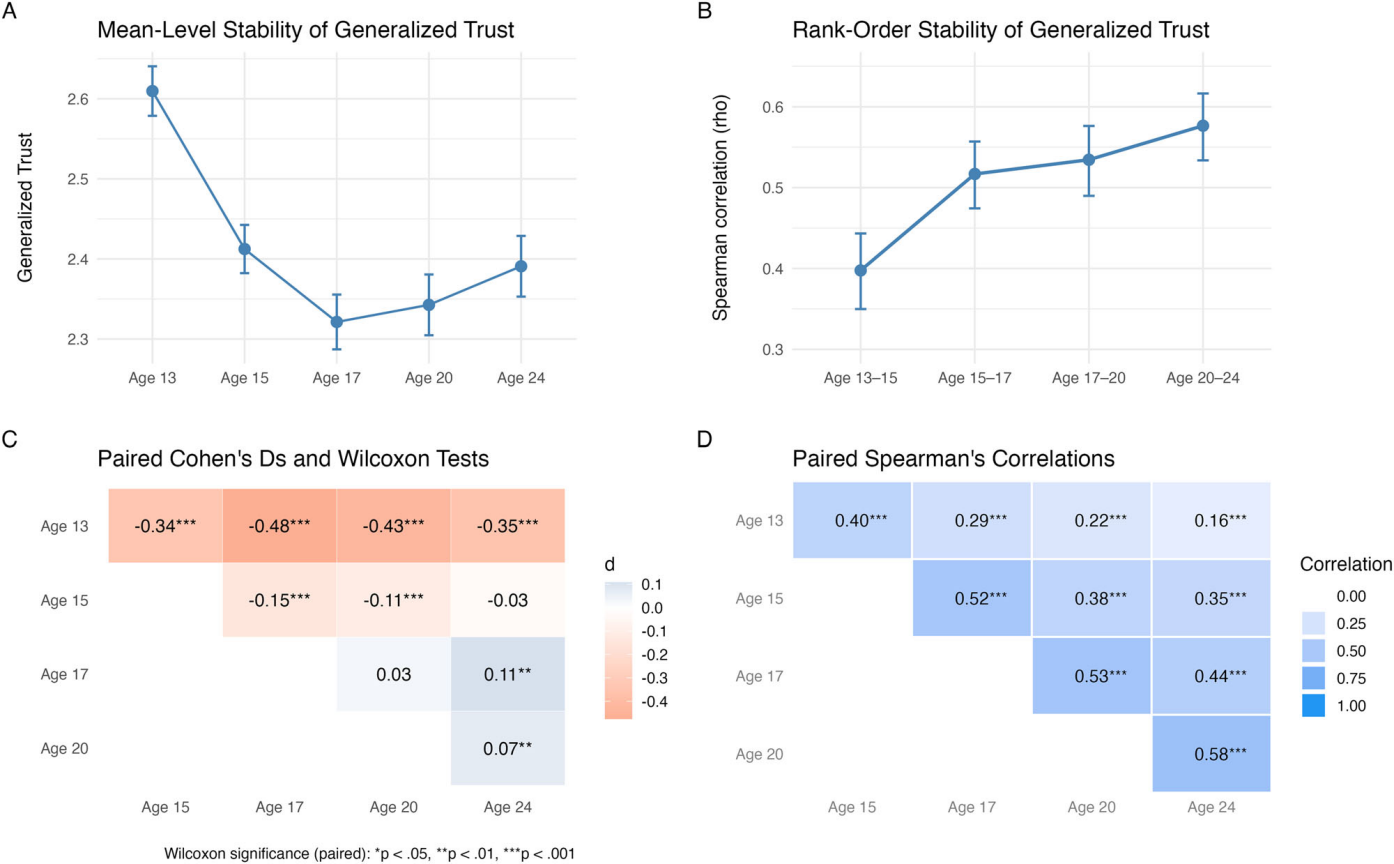
Developmental course and stability of trust. **A** Mean-level stability of generalized trust from age 13 to age 24; age-specific means and their corresponding 95% Confidence Interval (CI) are displayed. **B** Rank-order stability of generalized trust from age 13 to age 24, Spearman correlations and their corresponding 95% CI are depicted. **C** Heat map of paired Cohen’s Ds and Wilcoxon tests between all measurement timepoints is displayed. **D** Correlation matrix of bivariate Spearman correlations between all measurement occasions is displayed. All analyses were conducted with available data (see Suppl. Fig. 2).**A** Means and 95% CIs are based on *n* = 1360, 1444, 1302, 1180, and 1159 participants at ages 13, 15, 17, 20, and 24, respectively. Pairwise effect sizes and correlations use complete pairs with *n* = 1323 (13–15), 1207 (13–17), 1101 (13–20), 1083 (13–24), 1283 (15–17), 1164 (15–20), 1142 (15–24), 1115 (17–20), 1094 (17–24), and 1064 (20–24) participants. Significance levels are indicated at: *р < .05, **p < 01, ***p < 001.

Descriptive analyses indicated that generalized trust followed a curvilinear trajectory with a moderate mean-level decline from age 13 to age 17 (Cohen’s *D*_age13–17_ = −0.48) and a small increase from age 17 to age 24 (Cohen’s *D*_age17–24_ = 0.11, see Fig. 1A, C). Rank-order stability increased across adolescence (Age 13–15: Spearman’s rho = 0.40, 95%-CI [0.35, 0.44], *p* < 0.001, to Age 20–24: rho = 0.58, 95%-CI [0.53, 0.62], *p* < 0.001), with the largest increases in early- to mid-adolescence (see Fig. 1B, D). Longitudinal LMMs identified the best-fitting model as one including fixed linear and quadratic age terms, a random slope for linear age, and a random intercept for participant ID (see Supplementary Table 2 and 3). The model demonstrated substantial explanatory power (conditional *R*^2^ = 0.49 while fixed effects alone accounted for a small proportion of variance (marginal *R*² = 0.03). Within this model, the effect of age was statistically significant and negative (*β* = −0.14, 95%-CI [−0.15, −0.12], *p* < 0.001), whereas the quadratic age term was statistically significant and positive (*β* = 0.01, 95%-CI [0.01, 0.01], *p* < 0.001). Random effects indicated considerable variance in intercepts across individuals (τ00 = 0.38) and minimal variance in age slopes (τ11 = 0.01), with a negative correlation between intercepts and slopes (ρ01 = –0.26).

### Sociodemographic and family-level childhood predictors of generalized trust

**Fig. 2:**
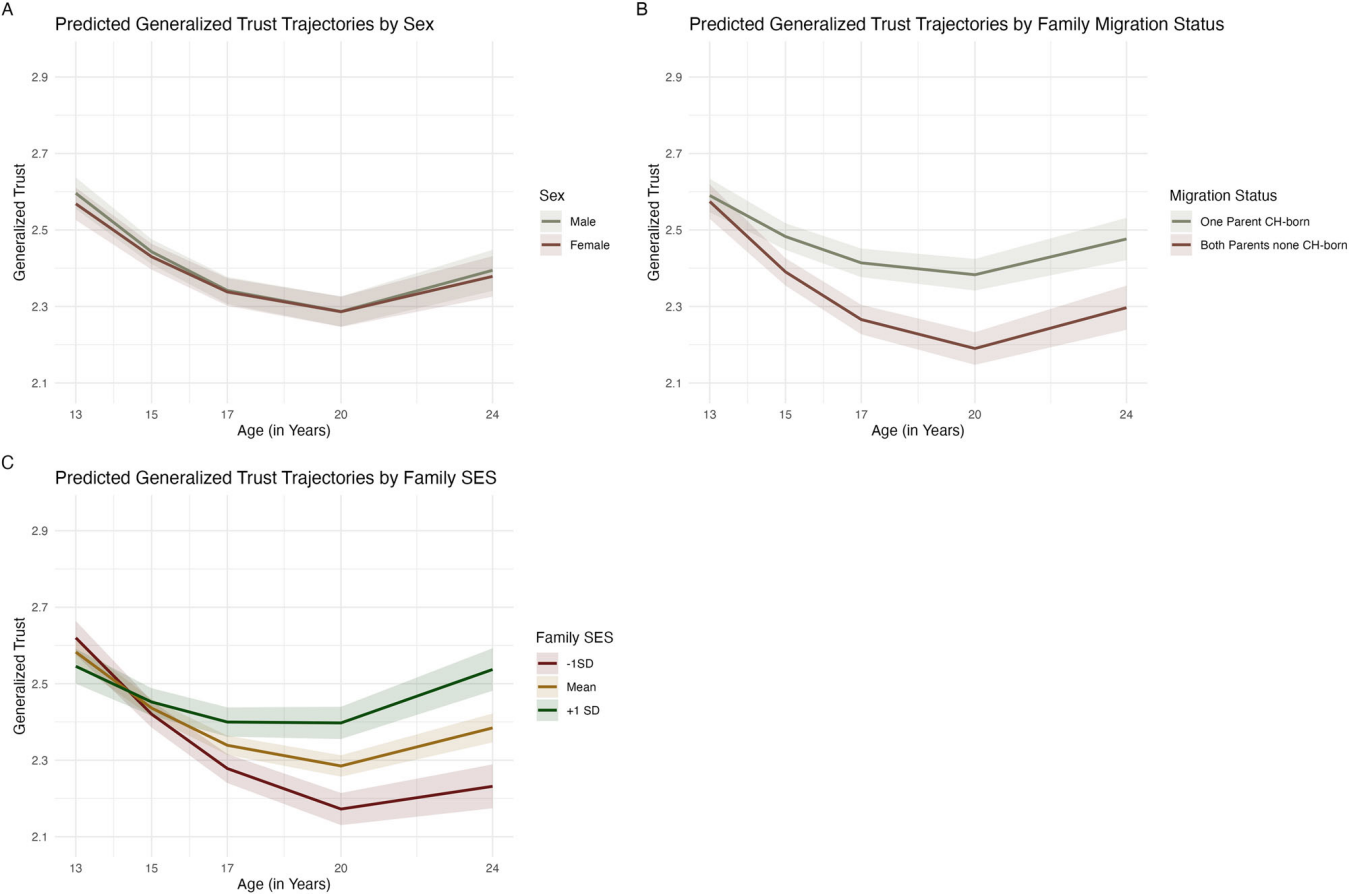
Sociodemographic predictors of generalized trust trajectories. All plots depict estimated marginal means of observed generalized trust for specific predictors across ages from predictor × age interaction models, adjusting for all other covariates in the model. **A**, **B**, and **C** are based on Model 2 in Supplementary Table 4. Plots were created based on single models fitted on merged datasets after multiple imputation (*n* = 1481). We used observed trust as the outcome, whereas Supplementary Table 4 used standardized scores. For model estimation, age was coded as years since baseline (0, 2, 4, 7, 11) and then mapped back to chronological age (13, 15, 17, 20, 24 years) for plotting. Shaded error bands depict the 95% CI of each model-predicted mean.

Expanding the baseline developmental model, we included sex, migration background, and family SES as time-invariant fixed effects and added interaction terms with age and age^2^ (see Supplementary Table 4 and interaction models in Fig. 2). These models showed that higher family SES was associated with higher levels of generalized trust, whereas having a migration background was linked to lower trust. Sex did not significantly predict trust levels (see Supplementary Table 4, Model 1). In models including sociodemographic × age interactions, significant interaction terms were found for both family SES and migration background with age and age^2^ (see Supplementary Table 4, Model 2). Visualizations of these models show that trust disparities related to SES and migration background were minimal at age 13 but widened throughout adolescence (see Fig. 2A–C). In exploratory, descriptive analyses incorporating participants’ highest educational attainment at age 24, generalized trust was higher among those with a baccalaureate, vocational tertiary education, or academic tertiary education than among those without secondary education or an apprenticeship (see Supplementary Table 5). The education  × age interaction suggests that trust trajectories diverge over time, with disparities opening alongside diverging educational attainments.

**Fig. 3:**
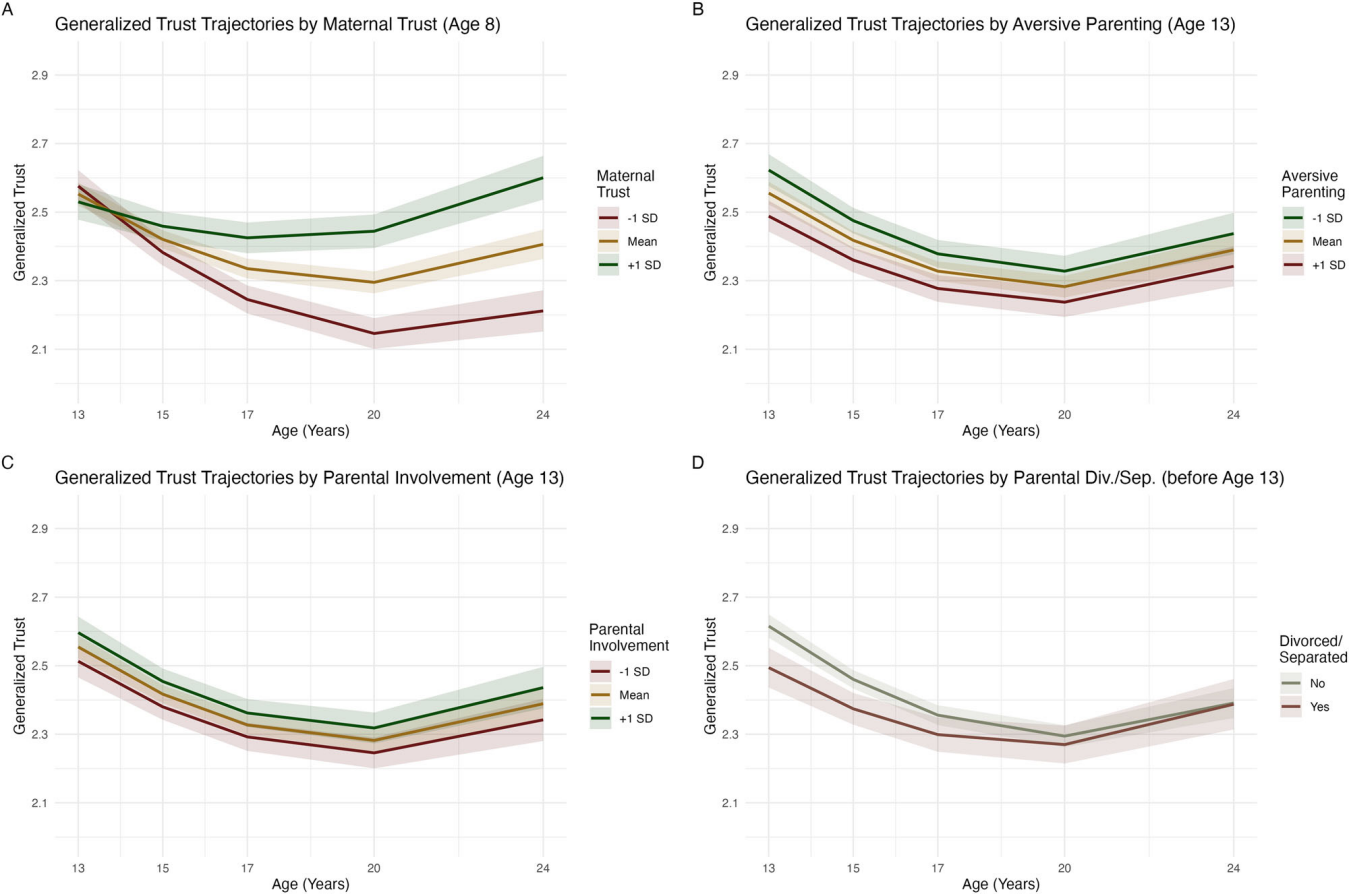
Family-level predictors of generalized trust trajectories. All plots depict estimated marginal means of observed generalized trust for specific predictors across ages from predictor × age interaction models, adjusting for all other covariates in the model. Plots **A**–**D** are based on Model 4 in Supplementary Table 4. Plots were created based on single models fitted on multiply imputed and merged datasets (*n* = 1481; see Suppl. Methods 1); we used observed trust as the outcome for descriptive purposes in the figures, whereas Supplementary Table 4 used standardized trust scores. For model estimation, age was coded as years since baseline (0, 2, 4, 7, 11) and then mapped back to chronological age (13, 15, 17, 20, 24 years) for plotting. Shaded error bands depict the 95% CI of each model-predicted mean.

Next, we extended the model by adding family-level predictors as time-invariant fixed effects in co-adjusted models (correlations matrix of all study variables included in Supplementary Fig. 1). Maternal generalized trust (age 8) and parental involvement (age 13) were positively associated with higher levels of trust, whereas parental divorce before age 13 and aversive parenting at age 13 were linked to lower trust, adjusting for sociodemographics (see Supplementary Table 4, Model 3). When family-level predictors were included, the effect of adolescent family SES on trust was no longer significant, while the migration effect remained robust. Interaction models showed significant maternal trust × age interactions and marginally significant divorce × age interactions (see Supplementary Table 4, Model 4). Visualizations indicate that the effects of parental involvement and aversive parenting remained stable throughout adolescence, whereas the association of maternal trust with generalized trust increased over time, and the effect of parental divorce or separation diminished (see Fig. 3).

### Time-varying peer victimization predicting generalized trust

**Fig. 4:**
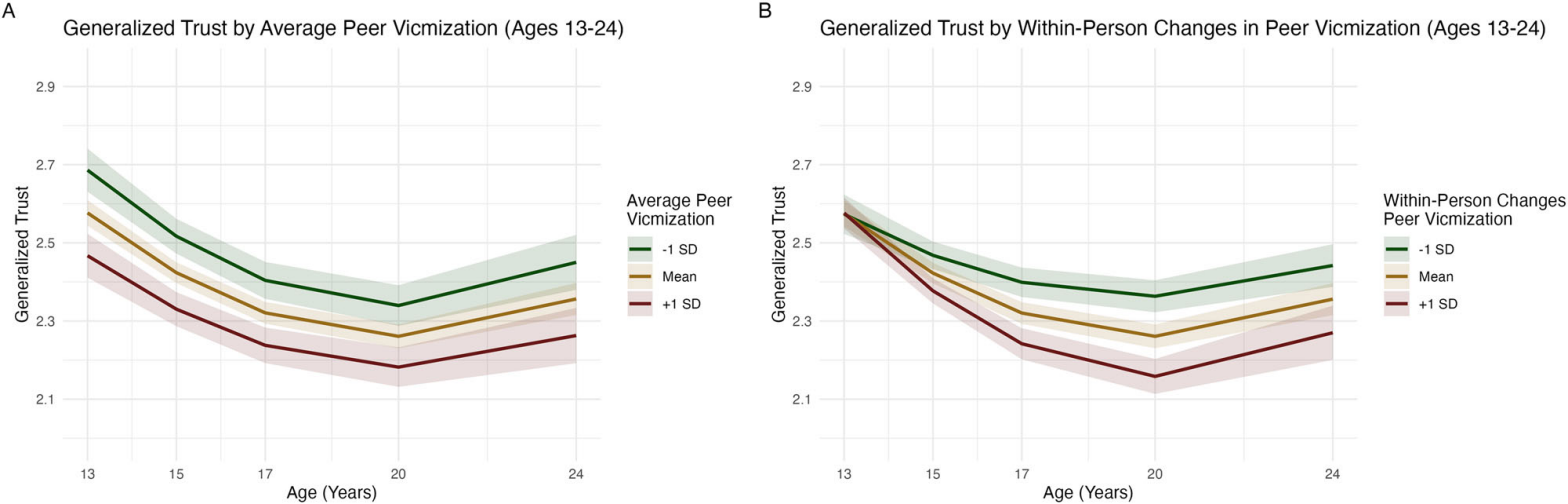
Between- and within-person levels of peer victimization as predictors of generalized trust trajectories. Plots depict estimated marginal means for between and within-person effects of peer victimization across ages from peer victimization × age interaction models, adjusting for all other covariates in the model. (**A**) and (**B**) are based on Supplementary Table 7. Plots were created based on single models fitted on multiply imputed and merged datasets (*n* = 1481; see Suppl. “Methods” 1). We used observed trust for descriptive purposes in the figures, whereas Supplementary Table 7 used standardized trust scores. For model estimation, age was coded as years since baseline (0, 2, 4, 7, 11) and then mapped back to chronological age (13, 15, 17, 20, 24 years) for plotting. Shaded error bands depict the 95% CI of each model-predicted mean.

Finally, we included time-varying peer victimization into the models by including both between-person and within-person effects, along with their interactions with age, and specified a random slope for within-person changes in peer victimization. The model that included the random effect for within-person peer victimization better fit the data than the model without (see Supplementary Table 6). The fixed-effects-only, both between-person and within-person peer victimization were significantly associated with lower levels of generalized trust across all measurement timepoints (see Supplementary Table 7, Model 5). Interaction models revealed that the within-person peer victimization effect significantly interacted with age, with the strongest negative associations with trust observed in late adolescence (ages 17 and 20; see Fig. 4A, B, and Supplementary Table 6, Model 6). Random slopes indicated variability in the relationship between within-person peer victimization and trust across individuals, suggesting that when participants experienced more peer victimization than usual, the magnitude of its impact on trust differed substantially between individuals (see Supplementary Table 7).

## Discussion

Our study investigated a broad set of multilevel predictors of curvilinear trust trajectories from adolescence to young adulthood using data from a cohort study spanning 11 years. Our findings reveal widening social disparities in generalized trust across adolescence, with higher family SES linked to greater trust and a migration background associated with lower trust. Family- and parenting-related factors significantly influenced generalized trust levels and partially shaped trust trajectories, with the effects of maternal trust increasing and the effects of parental divorce or separation diminishing throughout adolescence. Peer victimization consistently predicted lower trust, with within-person changes exerting the strongest effects during late adolescence and young adulthood. Because our three-item scale primarily captures generalized interpersonal trust, our conclusions apply to this broad form of trust and not to more situational or target-specific forms of trust (e.g., in-group vs. out-group or experimental trust games). Together, these findings provide nuanced insights into the developmental course of generalized trust and highlight the sociodemographic, family-level and peer-victimization related factors that shape trust throughout adolescence.

### Developmental course and stability of generalized trust

We found a quadratic pattern in generalized trust, with a marked decline from early adolescence (age 13) to mid-adolescence (age 17), followed by a small increase into young adulthood (ages 20–24). Rank-order stability also increased across adolescence. These findings align with previous research from Sweden and the US, which similarly reported the steepest decline during early to mid-adolescence alongside rising rank-order stability^30,31,33^. Early adolescence is often characterized by increased social insecurity and exploration, as youth encounter a broader and more diverse social world^26,70^. During this formative period, exposure to varied environmental contexts, diverse interpersonal encounters, and negative social experiences can affect trust levels^30,31,33^. Additionally, adolescents’ growing capacity for abstract thinking and differentiation between trust types (e.g., particularized vs. generalized trust) may lead them to become more adept at evaluating others’ trustworthiness and increase their skepticism^30,33^. In interpreting the decline in generalized trust between ages 13 and 17, it is also plausible that lower scores reflect adolescents becoming more realistic and cautious in their expectations of others, rather than simply less trusting. Likewise, the subsequent increase in trust between ages 17 and 24 may partly reflect movement into more selective environments (e.g., educational and vocational settings, close peer groups) in which interaction partners are, on average, considered more trustworthy. Taken together, our findings and prior evidence suggest that generalized trust is relatively high and malleable in early adolescence, becomes more calibrated and context-sensitive during mid-adolescence, and then increasingly stable as youth mature.

Interestingly, while trust levels in the Swedish sample returned to their initial levels by age 24, mean trust in the Swiss sample remained considerably lower than at age 13 (Cohen’s *D*_age 13 to 24_ = −0.35). Previous cross-national research has identified country-level factors contributing to mean-level differences in trust^71^, yet it remains unclear whether developmental trajectories differ systematically by country and what mechanisms explain such variation. While the Swedish data came from an accelerated longitudinal study involving five age cohorts and a two-wave design conducted in a medium-sized city between 2010/2011 and 2012/2013, our study followed a single cohort across five timepoints in the urban region of Zurich between 2011 and 2022. Potential explanations for the smaller trust recovery observed in our study may include structural and cultural differences, as well as time-period-related socio-historical events such as the COVID-19 pandemic, the Russian war on Ukraine, and shifting policies and political debates around safety.

### Sociodemographic predictors of trust trajectories

Higher family socioeconomic status (SES) was associated with greater generalized trust, whereas a migration background was associated with lower trust. Importantly, these disparities were age-dependent, absent at age 13 but significantly pronounced by young adulthood. These findings extend prior evidence that family resources and migration history increasingly shape adolescents’ trust experiences as social contexts become more complex, underscoring adolescence as a critical period for the emergence of such disparities^32,38–40^. At age 13, all participants were enrolled in secondary school; however, the Swiss education system subsequently channels adolescents into distinct educational and vocational trajectories strongly influenced by family SES and migration background, potentially influencing trust development. Moreover, migration background may accelerate declines in trust during adolescence, as migrant youth often face greater discrimination and social exclusion, which undermine interpersonal trust^72–74^. After accounting for structural and behavioral family-level factors, the effect of family SES was no longer significant, suggesting partial mediation by parental behaviors, whereas the effect of migration remained robust. Last, we found no significant effect of sex, consistent with prior studies in adolescence^33,41^. In additional exploratory models, adolescents who later attained higher educational qualifications showed more favorable trust trajectories; however, our main inferences are based on models that do not condition on future educational attainment, and these exploratory analyses should be interpreted as describing differences by later educational pathway rather than implying that education causally shaped earlier trust levels. Together, these findings highlight the importance of contextual factors in shaping trust development from adolescence to young adulthood.

### Family-level predictors of trust trajectories

Family-level factors, including parental behaviors and family structure, were associated with trust trajectories, with varying magnitude across adolescence. Maternal generalized trust and parental involvement were positively associated with trust, consistent with findings that parents who model trusting, autonomy-supportive, and compassionate attitudes foster trust in their children through emotional safety and social learning^33,38,42^. In contrast, aversive parenting and parental divorce or separation were associated with lower trust, likely reflecting how these adverse experiences undermine emotional security, heighten vigilance, and hinder the formation of positive schemas about others’ trustworthiness^38,46^.

Interaction models revealed developmental dynamics the impact of maternal trust intensified across adolescence; parental involvement and aversive parenting exerted a consistently stable influence, and the negative association with parental divorce diminished over time. As adolescents mature and interpret social cues more autonomously, parental attitudes may become increasingly salient, with maternal distrust reinforcing cautious social schemas, particularly as youth seek independence and navigate diverse social environments^33,42,75^. Meanwhile, parental involvement and aversive parenting shape daily emotional safety throughout adolescence, providing a stable context for trust formation regardless of age^33^. The negative impact of parental divorce or separation (before age 13) exerted larger effects closer to the family disruption. These effects, however, may lessen as adolescents adapt, develop coping strategies, or form new support systems outside the family^76^. Together, these findings underscore the importance of sustained supportive relationships for trust development during adolescence.

### Time-varying peer victimization predicting trust trajectories

Our findings demonstrate that youth who experience more peer victimization, whether on average or in specific developmental periods, tend to have lower trust, after adjusting for sociodemographic and family-level factors. Importantly, beyond aggregate effects, within-person fluctuations in peer victimization had the strongest negative association with trust during late adolescence and young adulthood. These results align with prior research showing that peer victimization contributes to declines in generalized trust, by fostering social caution and negative social schemas^32,48–50^.

While peer victimization typically peaks in early- to mid-adolescence, late adolescence and young adulthood remain periods of heightened vulnerability and new opportunities for victimization, possibly due to developmental transitions, changing social contexts, and exposure to new environments^70,77^. Additionally, peer victimization in late adolescence and young adulthood may disproportionately involve individuals who experience persistent, chronic, or severe forms of victimization and related stigma.

Furthermore, variability in random slopes revealed that some adolescents were more strongly affected by increases in peer victimization, underscoring individual differences in how victimization experiences are internalized and reflected in trust. This supports previous evidence that not all adolescents respond similarly to interpersonal threats; rather, contextual factors and personal appraisals interact to shape adaption^48,49^. Together, these results highlight the need for future research to elucidate the specific developmental mechanisms, temporal sensitivities, and moderating factors underlying the effect of time-varying peer victimization on trust trajectories.

### Cultural and experiential perspectives on trust development

Together, our findings support a more integrative view of cultural and experiential perspectives on generalized trust. Rather than suggesting a fully fixed, early-instilled cultural disposition that produces stable differences between cultural or ethnic groups, the developmental pattern indicates that cultural and socioeconomic embeddedness shape trust through cumulatively stratified experiences across adolescence and young adulthood. In this sense, migration background and SES can be understood as cultural and structural positions that channel adolescents into different opportunity structures and exposure to adversity. Robust links between proximal adversities and within-person changes in trust further support experiential accounts that emphasize ongoing plasticity. Thus, generalized trust appears neither purely a stable cultural trait nor merely situational, but rather emerges from dynamic interactions among early socialization, structurally patterned experiences, and contemporaneous exposures across development.

### Critical appraisal of findings

Although various sociodemographic, family-level or peer-related factors were significantly associated with generalized trust, effect sizes were modest (see marginal *R*^2^ values below 10% and conditional *R*^2^ values between 45–50%, in Supplementary Tables 2, 6, 7). This suggests that most variance in trust reflects stable, person-level factors and that the influence of environmental exposure may be constrained. Importantly, however, low malleability of trust may also result from limited change in environmental circumstances. Nevertheless, the findings highlight meaningful developmental patterns of vulnerability. Furthermore, distrust can be a healthy, adaptive response to chronic adversity; however, if it persists after the threat has passed, it may hinder positive social interactions in safer environments^75^. Thus, while high trust is generally viewed as beneficial, a certain degree of skepticism may serve important protective functions such as avoiding exploitation or harm^23^. Accordingly, trust development involves balancing openness to others with discernment about when and where distrust is warranted.

### Strengths and limitations

Major strengths of this study include its longitudinal design, which captured trust development across five timepoints over an 11-year period, enabling robust analysis of both change and stability from adolescence into young adulthood. The inclusion of a broad set of multilevel predictors and the use of hierarchical modeling allowed for rigorous adjustment of key confounders. Modeling quadratic growth trajectories further facilitated a nuanced understanding of when and how disparities in trust arise, contributing valuable insights into the complexity of social and developmental influences on trust over time.

Several limitations warrant consideration. First, trust was measured using a brief three-item scale that primarily indexes generalized interpersonal trust toward most others, which may constrain the ability to capture multidimensional aspects of trust such as situational (context-dependent) trust, trust in specific targets (e.g., peers, institutions), or in-group versus out-group trust. As a result, our findings cannot be readily generalized to more specific or behavioral manifestations of trust, which for other forms may follow different developmental courses^29^. Moreover, age-related differences in mean-level generalized trust should be understood as shifts in broad generalized expectations that integrate cumulative experience and developmental changes in social information processing, rather than as precise indices of context-specific or short-term trust dynamics. Trust should be acknowledged as a partly adaptive mechanism: low trust may be highly functional in certain contexts, and caution against oversimplistic and linear narratives about trust is therefore warranted. Second, the study did not employ dynamic models capable of examining bidirectional, time-lagged interactions between peer victimization and trust, limiting insights into potential feedback loops and causal processes across development. Third, the survey mode changed from paper-and-pencil to computer-based/online administration and may have influenced mean-level reports. Although generalized trust is relatively non-sensitive and prior work suggests only small mode effects even for sensitive topics^78,79^, we cannot fully exclude mode-related bias, and observed developmental trends should therefore be interpreted with some caution. Fourth, although between-school variance in trust remained relatively small, the slightly higher ICCs in young adulthood may indicate that early school and neighborhood environments contribute indirectly to the later stratification of generalized trust. We did not include schools as a separate model level, however, because the small school-level ICCs and the limited number of clusters would likely have introduced estimation challenges and substantially complicated the interpretation of SES and migration background effects. Lastly, generalized trust was first measured at age 13 and family-level predictors were only assessed from mid-childhood onward, missing earlier trust measures and exposures. Thus, while disparities in trust seem to emerge after age 13, this design cannot fully address the origins or earlier developmental antecedents of trust trajectories.

### Implications

These findings carry several important implications: For research, future studies should build on these findings by testing more mechanistic models that examine how childhood risk and protective factors, as well as proximate exposures, contribute to changes in generalized trust. Future work should employ more comprehensive, multidimensional trust measures that distinguish between generalized and particularized trust and trust in specific targets (e.g., peers, institutions), and should integrate behavioral paradigms (e.g., trust games) to capture situational and context-dependent aspects of trust. Research should also examine how sociodemographic factors (e.g., low SES or migration background) interact with specific parental behaviors and peer experiences to shape trust across development.

From an applied perspective, our results tentatively suggest that early adolescence may be a key period during which generalized trust is particularly malleable. Family, school, and community programs that foster fairness, compassion, and inclusion and reduce victimization could, in principle, be especially impactful when delivered during this period. Importantly, social contexts should not only prevent harmful interpersonal experiences but also provide repeated, concrete opportunities to experience others as reliable, fair, and cooperative in everyday interactions. Furthermore, although not tested in this study, broader approaches such as supporting civic engagement, strengthening institutional trust, and promoting strong informal social ties may help promote individual well-being and more resilient societies^80–82^.

## Conclusion

This study demonstrates that while generalized trust becomes increasingly stable over time, it is shaped by a range of risk and protective factors, including socioeconomic and migration background, parental trust, parenting practices, and peer victimization, with effects that vary by age. These findings highlight adolescence as a sensitive period for trust formation and the need for developmentally timed, multilevel interventions to foster appropriate trust, especially among young people who have not had the opportunity to experience trust-promoting family and peer environments.

## Supplementary information

Transparent Peer Review file

Supplementary Information

## Data Availability

All data used within this study are available through SWISSUbase (https://www.swissubase.ch/de/catalogue/studies/9707/20382/overview^68,69^). All users are required to sign a user agreement with the University of Zurich or with SWISSUbase to access the data.

## Code availability

The R code used for data preparation, multiple imputation, statistical analyses, and figure generation in this study is available in the repository “Family background and peer victimization are associated with generalized trust from adolescence to adulthood” on OSF. The repository contains all analysis scripts and information on software dependencies (R version and package versions). The code can be accessed at 10.17605/OSF.IO/PC9HB.

## References

[1] Uslaner, E. M. *in T**he Oxford Handbook of Social and Political Trust.*3–13 (Oxford Academic, 2018).

[2] Thielmann, I. & Hilbig, B. E. Trust: an integrative review from a person–situation perspective. *Rev. Gen. Psychol.* **19**, 249–277 (2015).

[3] Hamamura, T., Li, L. M. W. & Chan, D. The association between generalized trust and physical and psychological health across societies. *Soc. Indic. Res.* **134**, 277–286 (2016).

[4] Sheridan, M. A. & Zeanah, C. H. Dimensions of adversity exposure and psychopathology: deprivation and threat. *J. Am. Acad. Child Adolesc. Psychiatry* **58**, S339–S340 (2019).

[5] Miller, A. B. et al. Dimensions of deprivation and threat, psychopathology, and potential mediators: a multi-year longitudinal analysis (vol 127, pg 160, 2018). *J. Abnorm. Psychol.* **129**, 363–363 (2020). 29528670 10.1037/abn0000331 PMC5851283

[6] O’Donovan, A., Slavich, G. M., Epel, E. S. & Neylan, T. C. Exaggerated neurobiological sensitivity to threat as a mechanism linking anxiety with increased risk for diseases of aging. *Neurosci. Biobehav. Rev.* **37**, 96–108 (2013). 23127296 10.1016/j.neubiorev.2012.10.013 PMC4361087

[7] Slavich, G. M. Social safety theory: a biologically based evolutionary perspective on life stress, health, and behavior. *Annu. Rev. Clin. Psychol.* **16**, 265–295 (2020). 32141764 10.1146/annurev-clinpsy-032816-045159 PMC7213777

[8] Graves, S. B. & Larkin, E. Lessons from Erikson. *J. Intergenerat.Relatsh.* **4**, 61–71 (2006).

[9] Bowlby, J. in *Influential Papers from the 1950s*. 222–273 (Routledge, 1958).

[10] Sameroff, A. A unified theory of development: a dialectic integration of nature and nurture. *Child Dev.* **81**, 6–22 (2010). 20331651 10.1111/j.1467-8624.2009.01378.x

[11] Osher, D., Cantor, P., Berg, J., Steyer, L. & Rose, T. Drivers of human development: how relationships and context shape learning and development. *Appl. Dev. Sci.* **24**, 6–36 (2018).

[12] Werner, E. E. Resilience in development. *Curr. Direct. Psychol. Sci.* **4**, 81–84 (1995).

[13] Werner, E. E. Risk, resilience, and recovery: perspectives from the Kauai Longitudinal Study. *Dev. Psychopathol.* **5**, 503–515 (1993).

[14] Bellis, M. A., Hughes, K., Ford, K., Sharp, C. & Hill, R. Associations between adverse childhood experiences and trust in health and other information from public services, professionals and wider sources: national cross sectional survey. *BMJ Public Health* **2**, e000868 (2024). 40018105 10.1136/bmjph-2023-000868 PMC11812900

[15] Panter-Brick, C. & Leckman, J. F. Editorial Commentary: Resilience in child development-interconnected pathways to wellbeing. *J. Child Psychol. Psychiatry* **54**, 333–336 (2013). 23517424 10.1111/jcpp.12057

[16] Birkhäuer, J. et al. Trust in the health care professional and health outcome: a meta-analysis. *PLoS One* **12**, e0170988 (2017). 28170443 10.1371/journal.pone.0170988 PMC5295692

[17] Colquitt, J. A., Scott, B. A. & LePine, J. A. Trust, trustworthiness, and trust propensity: a meta-analytic test of their unique relationships with risk taking and job performance. *J. Appl Psychol.* **92**, 909–927 (2007). 17638454 10.1037/0021-9010.92.4.909

[18] Jones, S. L. & Shah, P. P. Diagnosing the locus of trust: a temporal perspective for trustor, trustee, and dyadic influences on perceived trustworthiness. *J. Appl Psychol.* **101**, 392–414 (2016). 26348476 10.1037/apl0000041

[19] Glanville, J. L. & Paxton, P. How do we learn to trust? A confirmatory tetrad analysis of the sources of generalized trust. *Soc. Psychol. Q.* **70**, 230–242 (2007).

[20] Krabbendam, L., Sijtsma, H., Crone, E. A. & van Buuren, M. Trust in adolescence: development, mechanisms and future directions. *Dev. Cogn. Neurosci.* **69**, 101426 (2024). 39121641 10.1016/j.dcn.2024.101426 PMC11363724

[21] Mayer, R. C., Davis, J. H. & Schoorman, F. D. An integrative model of organizational trust. *Acad. Manag. Rev.* **20**, 709–734 (1995).

[22] Kettlewell, N. & Tymula, A. Heritability across different domains of trust. *J. Econ. Behav. Organ.* **219**, 549–563 (2024).

[23] Van Lange, P. A. M. Generalized trust. *Curr. Direct. Psychol. Sci.* **24**, 71–76 (2015).

[24] Dawson, C. How persistent is generalised trust? *Sociology* **53**, 590–599 (2019).

[25] Uslaner, E. M. Where you stand depends upon where your grandparents sat: the inheritability of generalized trust. *Public Opin. Q.* **72**, 725–740 (2008).

[26] Sawyer, S. M., Azzopardi, P. S., Wickremarathne, D. & Patton, G. C. The age of adolescence. *Lancet Child Adolesc. Health* **2**, 223–228 (2018). 30169257 10.1016/S2352-4642(18)30022-1

[27] Arnett, J. J. Emerging adulthood: a theory of development from the late teens through the twenties. *Am. Psychol.* **55**, 469–480 (2000). 10842426

[28] Sijtsma, H. et al. The development of adolescent trust behavior. *J. Exp. Child Psychol.* **231**, 105653 (2023). 36848696 10.1016/j.jecp.2023.105653

[29] Reiter, A. M. F. et al. Self-reported childhood family adversity is linked to an attenuated gain of trust during adolescence. *Nat. Commun.* **14**, 6920 (2023). 37903767 10.1038/s41467-023-41531-z PMC10616102

[30] Flanagan, C. A. & Stout, M. Developmental patterns of social trust between early and late adolescence: age and school climate effects. *J. Res. Adolesc.* **20**, 748–773 (2010). 20936077 10.1111/j.1532-7795.2010.00658.x PMC2950022

[31] Abdelzadeh, A. & Lundberg, E. Solid or flexible? Social trust from early adolescence to young adulthood. *Scand. Polit. Stud.* **40**, 207–227 (2017).

[32] Lundberg, E. & Abdelzadeh, A. The role of school climate in explaining changes in social trust over time. *Scand. J. Educ. Res.* **63**, 712–724 (2018).

[33] Wray-Lake, L. & Flanagan, C. A. Parenting practices and the development of adolescents’ social trust. *J. Adolesc.* **35**, 549–560 (2012). 22015215 10.1016/j.adolescence.2011.09.006 PMC11378488

[34] Masten, A. S., Lucke, C. M., Nelson, K. M. & Stallworthy, I. C. Resilience in development and psychopathology: multisystem perspectives. *Annu Rev. Clin. Psychol.* **17**, 521–549 (2021). 33534615 10.1146/annurev-clinpsy-081219-120307

[35] Ungar, M. & Theron, L. Resilience and mental health: how multisystemic processes contribute to positive outcomes. *Lancet Psychiatry* **7**, 441–448 (2020). 31806473 10.1016/S2215-0366(19)30434-1

[36] Bronfenbrenner, U. Developmental research, public policy, and the ecology of childhood. *Child Dev.* **45**, 1–5 (1974).

[37] Lopez, M. et al. The social ecology of childhood and early life adversity. *Pediatr. Res.* **89**, 353–367 (2021). 33462396 10.1038/s41390-020-01264-x PMC7897233

[38] Kim, Y. I., VanderWeele, T. J. & Johnson, B. R. Childhood predictors of perceptions of social trust across 22 countries in the global flourishing study. *Sci. Rep.* **15**, 14358 (2025). 40307267 10.1038/s41598-024-78201-z PMC12043862

[39] Alesina, A. & La Ferrara, E. Who trusts others? *J. Public Econ.* **85**, 207–234 (2002).

[40] Hamamura, T. Social class predicts generalized trust but only in wealthy societies. *J. Cross-Cult. Psychol.* **43**, 498–509 (2012).

[41] Janmaat, J. G., The development of generalized trust among young people in England. *Soc. Sci*. **8**. 10.3390/socsci8110299. (2019).

[42] Stolle, D. & Nishikawa, L. Trusting others—how parents shape the generalized trust of their children. *Comp. Sociol.* **10**, 281–314 (2011).

[43] King, V. Parental divorce and interpersonal trust in adult offspring. *J. Marriage Fam.* **64**, 642–656 (2002).

[44] Pitula, C. E., Wenner, J. A., Gunnar, M. R. & Thomas, K. M. To trust or not to trust: social decision*-*making in post*-*institutionalized, internationally adopted youth. *Dev. Sci.* **20**, e12375 (2017). 10.1111/desc.12375 PMC5069074 27089448

[45] Giulietti, C., Rettore, E. & Tonini, S. The chips are down: the influence of family on children’s trust formation. *J. Popul. Econ.* **36**, 211–233 (2022).

[46] Brown, D. Childhood experiences, growing up “in care,” and trust: a quantitative analysis. *Child. Youth Serv. Rev*. **144**. 10.1016/j.childyouth.2022.106734 (2023).

[47] Scholte, R. H. & Van Aken, M. A. in *Handbook of Adolescent Development*. 175–199 (Psychology Press, 2020).

[48] Tsomokos, D. I. & Slavich, G. M. Bullying fosters interpersonal distrust and degrades adolescent mental health as predicted by Social Safety Theory. *Nat. Ment. Health* **2**, 328–336 (2024). 38682098 10.1038/s44220-024-00203-7 PMC11052587

[49] Betts, L. R., Houston, J. E., Steer, O. L. & Gardner, S. E. Adolescents’ experiences of victimization: the role of attribution style and generalized trust. *J. Sch. Violence* **16**, 25–48 (2016).

[50] Zhao, Y., Ji, X., Feng, N. & Cui, L. Longitudinal relationships between bullying victimization and depressive symptoms and the mediating role of interpersonal trust in middle adolescents. *J. Youth Adolesc.* **54**, 2681–2694 (2025). 40759844 10.1007/s10964-025-02231-7

[51] Brown, B. B. & Larson, J. in *Handbook of Adolescent Psychology* (John Wiley & Sons, Inc.,2009).

[52] Ribeaud, D., Murray, A., Shanahan, L., Shanahan, M. J. & Eisner, M. Cohort profile: the Zurich Project on the Social Development from Childhood to Adulthood (z-proso). *J. Dev. Life Course Criminol.* **8**, 151–171 (2022). 35223378 10.1007/s40865-022-00195-x PMC8860297

[53] Steinhoff, A., Bechtiger, L., Ribeaud, D., Eisner, M. & Shanahan, L. Self-, other-, and dual-harm during adolescence: a prospective-longitudinal study of childhood risk factors and early adult correlates. *Psychol. Med.* **53**, 3995–4003 (2023). 35297361 10.1017/S0033291722000666 PMC10317800

[54] z-proso Project Team. *z-proso Handbook: Instruments and Procedures in the Adolescent and Young Adult Surveys (Age 11 to 20; Waves K4-K9)-Short Version*. (University of Zurich, Zurich, 2024).

[55] Delhey, J., Newton, K. & Welzel, C. How general is trust in “Most People”? Solving the radius of trust problem. *Am. Sociol. Rev.* **76**, 786–807 (2011).

[56] Smith, S. S. Race and trust. *Annu. Rev. Sociol.* **36**, 453–475 (2010).

[57] Inglehart, R. et al. World Values Survey: Round One - Country-Pooled Datafi le. Madrid, Spain & Vienna, Austria: JD Systems Institute & WVSA Secretariat. http://www.worldvaluessurvey.org/WVSDocumentationWV1.jsp (2018).

[58] Nivette, A., Eisner, M. & Ribeaud, D. Developmental predictors of violent extremist attitudes. *J. Res. Crime. Delinquency* **54**, 755–790 (2017).

[59] Ganzeboom, H. B., De Graaf, P. M. & Treiman, D. J. A standard international socio-economic index of occupational status. *Soc. Sci. Res.* **21**, 1–56 (1992).

[60] z-proso Project Team. *z-proso Handbook: Instruments and Procedures in the Parent Surveys(Waves P1-P4; 2004–**2008), Version 1.1.0*. (University of Zurich, 2025).

[61] Shelton, K. K., Frick, P. J. & Wootton, J. Assessment of parenting practices in families of elementary school-age children. *J. Clin. Child Psychol.* **25**, 317–329 (1996).

[62] Murray, A. L. et al. Validation of a brief self-report measure of adolescent bullying perpetration and victimization. *Assessment* **28**, 128–140 (2021). 31280595 10.1177/1073191119858406

[63] Powell, M. J. *The BOBYQA Algorithm for Bound Constrained Optimization without Derivatives. Cambridge NA Report NA2009/06*.Vol. 26, 26–46 (University of Cambridge, Cambridge, 2009)

[64] McCormick, E. M., Byrne, M. L., Flournoy, J. C., Mills, K. L. & Pfeifer, J. H. The Hitchhiker’s guide to longitudinal models: a primer on model selection for repeated-measures methods. *Dev. Cogn. Neurosci.* **63**, 101281 (2023). 37536082 10.1016/j.dcn.2023.101281 PMC10412784

[65] Rubin, D. B. in *Flexible Imputation of Missing Data, Second Edition*. 29–62 (Chapman and Hall/CRC, 2018).

[66] Lüdecke, D., Ben-Shachar, M., Patil, I., Waggoner, P. & Makowski, D. Performance: an R package for assessment, comparison and testing of statistical models. *J. Open Source Softw*. **6**. 10.21105/joss.03139 (2021).

[67] RStudio, T. *RStudio: Integrated Development for R*. http://www.rstudio.com (R Studio, Inc., Boston, MA, 2015).

[68] Eisner, M. et al. *z-proso: Adolescent and Young Adult Surveys (Age 11 to 24; Waves K4–K9)*. (FORS, Lausanne, 2025).

[69] Eisner, M., Ribeaud, D. & Shanahan, L. *z-proso: Parents Interviews at Focal Participant Ages 7 to 11 (Waves P1-P4)*. (FORS, Lausanne, 2025).

[70] Arnett, J. J. *Emerging Adulthood: the Winding Road from the Late Teens through the Twenties*. (Oxford University Press, 2023)

[71] Delhey, J. & Newton, K. Social trust: global pattern or Nordic exceptionalism? *WZB Discussion Paper. (Wissenschaftszentrum Berlin für Sozialforschung (WZB)**,* Berlin, 2004).

[72] Ljunge, M. Trust issues: Evidence on the intergenerational trust transmission among children of immigrants. *J. Econ. Behav. Organ.* **106**, 175–196 (2014).

[73] Borho, A., Morawa, E., Schug, C. & Erim, Y. Perceived post-migration discrimination: the perspective of adolescents with migration background. *Eur. Child Adolesc. Psychiatry* **32**, 2427–2438 (2023). 36127567 10.1007/s00787-022-02084-6 PMC10682162

[74] Delaruelle, K. et al. Mental health in adolescents with a migration background in 29 European countries: the buffering role of social capital. *J. Youth Adolesc.* **50**, 855–871 (2021). 33791946 10.1007/s10964-021-01423-1

[75] Slavich, G. M. et al. Social safety theory: conceptual foundation, underlying mechanisms, and future directions. *Health Psychol. Rev.* **17**, 5–59 (2023). 36718584 10.1080/17437199.2023.2171900 PMC10161928

[76] Cao, H., Fine, M. A. & Zhou, N. The Divorce Process and Child Adaptation Trajectory Typology (DPCATT) Model: the shaping role of predivorce and postdivorce interparental conflict. *Clin. Child Fam. Psychol. Rev.* **25**, 500–528 (2022). 35106699 10.1007/s10567-022-00379-3 PMC8805665

[77] López-Castro, L., Smith, P. K., Robinson, S. & Görzig, A. Age differences in bullying victimisation and perpetration: evidence from cross-cultural surveys. *Aggress. Violent Behav.* **73**. 10.1016/j.avb.2023.101888 (2023).

[78] Colasante, E. et al. Paper-and-pencil versus computerized administration mode: comparison of data quality and risk behavior prevalence estimates in the European School Survey Project on Alcohol and other Drugs (ESPAD). *PLoS One* **14**, e0225140 (2019). 31747446 10.1371/journal.pone.0225140 PMC6867655

[79] Lucia, S., Herrmann, L. & Killias, M. How important are interview methods and questionnaire designs in research on self-reported juvenile delinquency? An experimental comparison of Internet vs paper-and-pencil questionnaires and different definitions of the reference period. *J. Exp. Criminol.* **3**, 39–64 (2007).

[80] Van Ingen, E. & Bekkers, R. Generalized trust through civic engagement? Evidence from five national panel studies. *Polit. Psychol.* **36**, 277–294 (2015).

[81] Sønderskov, K. M. & Dinesen, P. T. Trusting the state, trusting each other? The effect of institutional trust on social trust. *Polit. Behav.* **38**, 179–202 (2015).

[82] Glanville, J. L., Andersson, M. A. & Paxton, P. Do social connections create trust? An examination using new longitudinal data. *Soc. Forces* **92**, 545–562 (2013).

