# Supplementary material for: Family background and peer victimization are associated with generalized trust from adolescence to adulthood
Source: Commun Psychol. 2026 May 22;4:116. doi: 10.1038/s44271-026-00474-z (PMC13473010; doi:10.1038/s44271-026-00474-z)
Supplement: Supplementary file 1 [file 44271_2026_474_MOESM1_ESM.pdf]

nature portfolio

Peer Review File

Family background and peer victimization are associated with generalized trust from adolescence to adulthood

Corresponding Author: Dr David Bürgin

This file contains all editorial decision letters in order by version, followed by all author rebuttals in order by version.

Version 0:

Decision Letter:

** Please ensure you delete the link to your author homepage in this e-mail if you wish to forward it to your coauthors **

Dear Dr Bürgin,

Thank you for your patience during the peer-review process. Your manuscript titled "Developmental Trajectories and Social Determinants of Generalized Trust in Adolescence and Young Adulthood" has now been seen by 3 reviewers, and I include their comments at the end of this message. They find your work of interest but raised some important points. We are interested in the possibility of publishing your study in Communications Psychology, but would like to consider your responses to these concerns and assess a revised manuscript before we make a final decision on publication.

We therefore invite you to revise and resubmit your manuscript, along with a point-by-point response to the reviewers. Please highlight all changes in the manuscript text file.

Editorially, we consider it important that the revised manuscript addresses the modeling concerns raised by the reviewers, especially regarding educational attainment, clustering, between-person effects, and construct measurement.

As you revise the manuscript in response to these issues, please also implement all requests in the attached Mandatory Revision Requests document. All requirements listed in this document need to be fully met, or the work will be returned to you for further revisions without peer review. This workflow is in place to increase the likelihood that the paper will be accepted for publication. It reduces the number of rounds of revision (and review) and ensures that the reviewers vet a version of the article that is compliant with journal policies. If you have any questions regarding the required revisions, please contact the journal prior to resubmission to avoid a negative outcome.

We are committed to providing a fair and constructive peer-review process. Please don't hesitate to contact us if you wish to discuss the revision in more detail.

Please submit the following items:

- Revised manuscript
- Point-by-point response to the referees' comments
- Mandatory Revision Requests Table (attached).
- Cover letter (as a separate document)

via this link: Link Redacted .

** This url links to your confidential home page and associated information about manuscripts you may have submitted or

are reviewing for us. If you wish to forward this email to co-authors, please delete the link to your homepage first **

We hope to receive your revised paper within 8 weeks; please let us know if you aren't able to submit it within this time so that we can discuss how best to proceed. If we don't hear from you, and the revision process takes significantly longer, we may close your file. In this event, we will still be happy to reconsider your paper at a later date, provided it still presents a significant contribution to the literature at that stage.

We look forward to seeing the revised manuscript and thank you for the opportunity to review your work.

Best regards,

Jennifer Bellingtier

Jennifer Bellingtier, PhD
Senior Editor
Communications Psychology

REVIEWER EXPERTISE:
Developmental psychology, Trust, Longitudinal data modeling

REVIEWER REPORTS:

Reviewer #1 (Remarks to the Author):

This manuscript presents an original and methodologically rigorous longitudinal study examining generalized trust from adolescence into young adulthood, using data from the Zurich Project on Social Development from Childhood to Adulthood (n = 1,481). The study addresses a notable gap in developmental research on trust by providing valuable insights into mean-level trajectories, rank-order stability, and the roles of sociodemographic, family, and peer victimization factors. I have a few comments that the authors may wish to consider:

1. Given the brevity of the three-item measure, there may be limitations regarding content validity. Important facets of trust—such as situational versus generalized trust or in-group versus out-group trust—are not captured, which could constrain the interpretation of developmental trajectories and predictor associations. The authors briefly acknowledge this in the limitations section, but could they elaborate further on how this restricts the conclusions and how future research might address these gaps?

2. Given the clustered sampling of students within schools, it would be helpful for the authors to clarify whether school-level clustering was examined or to justify why such clustering was not modeled.

3. Using participants' educational attainment at age 24 to predict trust measured at earlier ages may raise concerns regarding reverse causality or overcontrol bias. The authors should justify this modeling decision, clarify the theoretical rationale, or consider alternative specifications.

4. Survey mode changes from paper-and-pencil to computer/online administration could influence observed mean-level differences. A brief discussion of potential mode effects would help contextualize the developmental trends.

5. Maternal trust is assessed using three binary items, which may limit reliability and reduce variability in estimates linking parental trust with adolescent trajectories. Discussing the reliability of this measure—and, more broadly, providing reliability estimates for all predictors—would enhance transparency.

Overall, this manuscript makes a meaningful contribution to understanding the development of generalized trust. Addressing these points might further enhance the interpretability of the findings.

Reviewer #2 (Remarks to the Author):

This manuscript describes a longitudinal study of the development of and the risk and protective factors of generalized trust from adolescence to young adulthood. The authors utilized longitudinal data collected from a large community sample of youth and their mothers who were living in Zurich, Switzerland, and incorporated a number of individual, interpersonal, and family-level risk and protective factors.

I appreciate the clear conceptualization of interpersonal trust and specifically generalized trust. However, it was odd to me to read the preceding paragraph on trust and child development (p. 3). To me, it would work better after the authors have provided the conceptual definition of trust.

In the intro, the authors compared the cultural and experiential perspectives of trust (p. 4). I would like to see the authors link their findings back to the two perspectives again more explicitly in the discussion.

In most of the intro, the authors provided rationale for examining different risk and protective factors of generalized trust. Could the authors speak more about the importance of examining the developmental trajectory of generalized trust in and of itself?

The authors reported random component of age ("minimal variance in age slopes…", p. 7). However, only linear age was allowed to randomly varying, but not quadratic age, is that correct?

Why did the authors incorporate highest education attainment at age 24 as a predictor of trust in their final set of analysis? Did the authors recode the time variable age? If not, this would be a decision that does not make a lot of sense to me conceptually.

The way the analysis was described gives me the impression that, although there was a considerable amount of variability in the intercept (i.e., age 17 trust, given how age was coded), the authors did not use time-invariant predictors to explain between-person differences in the intercept. Rather, they used time-invariant predictors in their level-1 model (in hierarchical linear modeling terminology). If this was not what the authors did, I would like to see some LMM equations in the supplemental materials to clear the confusion. If indeed the authors did not examine between-person differences at baseline generalized trust, could the authors please explain their rationale for not doing so?

Please provide more details about how the analytic sample was determined. Also, did the authors conduct any longitudinal attrition analysis?

Reviewer #3 (Remarks to the Author):

This is an excellent and well-written study on a very impressive dataset. The analyses are well conducted and the conclusions are well supported by the data. I have a couple of suggestions that are hopefully useful for the authors when revising the manuscript.

First and most important, I have some doubts whether the 3-item questionnaire actually measures generalized trust. Questions are: 'most people can be trusted', 'people usually try to help other people', 'most people try to be fair'. A declining score between ages 13-17 is interpreted as that adolescents become less trusting, but one could also argue that they become more realistic. We teach our adolescents that not everyone can be trusted (e.g. online, but also in real life). The increase again between ages 17-24 can also be related to more selective environments where individuals are actually more trustworthy. This is important for the framing of the study where it is assumed that more trust is always better.

Relatedly, the authors argue that there is no prior research on trust in adolescents, but there is actually a very large literature on these topics with more extensive trust-measuring questionnaires and trust games. I am not saying that this makes the current study less important: I believe these are data that should be published, but the authors could be more balanced with referring to prior developmental literature and the specific contribution of this study.

Finally, in the discussion the authors suggest that the solution for more generalized trust lies in civic engagement. But most of their results show that drops in generalized trust are related to societal encounters, so shouldn't the solution be also that our societies should provide more opportunities to trust others. This can possibly added for a balanced interpretation.

* TRANSPARENT PEER REVIEW: Communications Psychology uses a transparent peer review system. This means that we publish the editorial decision letters including Reviewers' comments to the authors and the author rebuttal letters online as a supplementary peer review file. However, on author request, confidential information and data can be removed from the published reviewer reports and rebuttal letters prior to publication. If your manuscript has been previously reviewed at another journal, those Reviewers' comments would not form part of the published peer review file.

** Visit Nature Research's author and referees' website at www.nature.com/authors for information about policies, services and author benefits**

Communications Psychology is committed to improving transparency in authorship. As part of our efforts in this direction, we are now requesting that all authors identified as 'corresponding author' create and link their Open Researcher and Contributor Identifier (ORCID) with their account on the Manuscript Tracking System prior to acceptance. ORCID helps the scientific community achieve unambiguous attribution of all scholarly contributions. You can create and link your ORCID from the home page of the Manuscript Tracking System by clicking on 'Modify my Springer Nature account' and following the instructions in the link below. Please also inform all co-authors that they can add their ORCIDs to their accounts and that

they must do so prior to acceptance.
https://www.springernature.com/gp/researchers/orcid/orcid-for-nature-research

For more information please visit http://www.springernature.com/orcid

If you experience problems in linking your ORCID, please contact the Platform Support Helpdesk.

Version 1:

Decision Letter:

** Please ensure you delete the link to your author homepage in this e-mail if you wish to forward it to your coauthors **

Dear Dr Bürgin,

Your manuscript titled "Emerging Disparities in Generalized Trust from Adolescence to Young Adulthood: Sociodemographic, Family, and Peer Influences" has now been seen by our reviewers, whose comments appear below. In light of their advice I am delighted to say that we are happy, in principle, to publish a suitably revised version in Communications Psychology.

We therefore invite you to revise your paper one last time to address the remaining concerns of our reviewers and a list of editorial requests. At the same time we ask that you edit your manuscript to comply with our format requirements and to maximise the accessibility and therefore the impact of your work.

EDITORIAL REQUESTS:

Please review our specific editorial comments and requests regarding your manuscript in the attached "Editorial Requests Table". Please outline your response to each request in the right hand column. Please upload the completed table with your manuscript files as a Related Manuscript file.

If you have any questions or concerns about any of our requests, please do not hesitate to contact me.

SUBMISSION INFORMATION:

In order to accept your paper, we require the files listed here https://www.nature.com/documents/commsj-file-checklist.pdf .

OPEN ACCESS:

Communications Psychology is a fully open access journal. Articles are made freely accessible on publication. For further information about article processing charges, open access funding, and advice and support from Nature Research, please visit https://www.nature.com/commspsychol/open-access

At acceptance, you will be provided with instructions for completing the open access licence agreement on behalf of all authors. This grants us the necessary permissions to publish your paper. Additionally, you will be asked to declare that all required third party permissions have been obtained, and to provide billing information in order to pay the article-processing charge (APC).

* TRANSPARENT PEER REVIEW: Communications Psychology uses a transparent peer review system. On author request, confidential information and data can be removed from the published reviewer reports and rebuttal letters prior to publication. If you are concerned about the release of confidential data, please let us know specifically what information you would like to have removed. Please note that we cannot incorporate redactions for any other reasons.

* CODE AVAILABILITY: All Communications Psychology manuscripts must include a section titled "Code Availability" at the end of the methods section. We require that the custom analysis code supporting your conclusions is made available in a publicly accessible repository at this stage; please choose a repository that generates a digital object identifier (DOI) for the code; the link to the repository and the DOI must be included in the Code Availability statement. Publication as Supplementary Information will not suffice.

* DATA AVAILABILITY:
All Communications Psychology manuscripts must include a section titled "Data Availability" at the end of the Methods section. More information on this policy, is available in the Editorial Requests Table and at http://www.nature.com/authors/policies/data/data-availability-statements-data-citations.pdf.

Please use the following link to submit the above items:

Link Redacted
** This url links to your confidential home page and associated information about manuscripts you may have submitted or be reviewing for us. If you wish to forward this email to co-authors, please delete the link to your homepage first **

We hope to hear from you within two weeks; please let us know if you need more time.

Best regards,

Jennifer Bellingtier

Jennifer Bellingtier, PhD
Senior Editor
Communications Psychology

REVIEWERS' EXPERTISE:
Developmental psychology, Trust, Longitudinal data modeling

REVIEWERS' COMMENTS:

Reviewer #1
No further comments for Authors.

Reviewer #2 (Remarks to the Author):

The authors have provided satisfactory clarifications and helpful additional information. I appreciate their efforts, particularly in explaining the modeling strategies and reintegrating cultural and experiential perspectives into the discussion. I enjoyed reading the responses and the revised manuscript, and I believe that the study makes a meaningful contribution to the field. I have no further comments.

Reviewer #3 (Remarks to the Author):

The authors have addressed all my comments in a satisfactory way. I enjoyed reading this strong manuscript.

** Visit Nature Research's author and referees' website at www.nature.com/authors for information about policies, services and author benefits**

Open Access This Peer Review File is licensed under a Creative Commons Attribution 4.0 International License, which permits use, sharing, adaptation, distribution and reproduction in any medium or format, as long as you give appropriate credit to the original author(s) and the source, provide a link to the Creative Commons license, and indicate if changes were made.
In cases where reviewers are anonymous, credit should be given to 'Anonymous Referee' and the source.
The images or other third party material in this Peer Review File are included in the article's Creative Commons license, unless indicated otherwise in a credit line to the material. If material is not included in the article's Creative Commons license and your intended use is not permitted by statutory regulation or exceeds the permitted use, you will need to obtain permission directly from the copyright holder.
To view a copy of this license, visit https://creativecommons.org/licenses/by/4.0/

Point-by-point Responses to the Reviewers

Reviewer #1 (Remarks to the Author):
This manuscript presents an original and methodologically rigorous longitudinal study examining generalized trust from adolescence into young adulthood, using data from the Zurich Project on Social Development from Childhood to Adulthood (n = 1,481). The study addresses a notable gap in developmental research on trust by providing valuable insights into mean-level trajectories, rank-order stability, and the roles of sociodemographic, family, and peer victimization factors.

Response R1.1: We sincerely thank the reviewer for their positive and encouraging evaluation of our manuscript.

I have a few comments that the authors may wish to consider:
1. Given the brevity of the three-item measure, there may be limitations regarding content validity. Important facets of trust—such as situational versus generalized trust or in-group versus out-group trust—are not captured, which could constrain the interpretation of developmental trajectories and predictor associations. The authors briefly acknowledge this in the limitations section, but could they elaborate further on how this restricts the conclusions and how future research might address these gaps?

Response R1.2: We appreciate this insightful comment. As we briefly introduced in the manuscript, interpersonal trust is conceptualized and measured differently across disciplines, ranging from behavioral trust assessed with game-theoretical paradigms (e.g., trust games), to particularized trust in specific others or groups (e.g., in-group versus out-group), to broad generalized trust in most unknown others. Following the reviewer's suggestion, we now clarify that our three-item measure is best understood as an indicator of generalized interpersonal trust and does not capture more situational or target-specific facets of trust (such as in-group vs. out-group or context-dependent trust). We therefore note that our conclusions pertain to developmental trajectories and predictors of generalized interpersonal trust, and should not be generalized to other forms of trust (e.g., trust behavior in experimental games, intergroup or community trust), for which different developmental patterns have been reported. The patterns we observed should be interpreted as changes in adolescents' broad expectations about „most people", rather than shifts in trust toward specific groups or contexts. Furthermore, we now highlight that future research should build on our findings by integrating multidimensional self-report instruments and experimental paradigms (e.g., trust games with different partners or groups) to better capture situational and group-specific aspects of trust development (see p. 7, p. 14, p.18-19).

2. Given the clustered sampling of students within schools, it would be helpful for the authors to clarify whether school-level clustering was examined or to justify why such clustering was not modeled.

Response R1.3: We thank the reviewer for raising this important point. In our study, students were initially clustered within schools; however, this clustering was effectively dissolved at the transition to upper secondary education between W4 and W5 due to changes in schools and tracks. To empirically assess the extent of school-level dependence, we estimated school-level ICCs for generalized trust at each wave and found very small between-school variance in adolescence (ICC range = 0.003–0.016) and still relatively small values in young adulthood (ICC range = 0.046–0.061), indicating that only a small share of variance was attributable to schools across the study period. In addition, our primary focus was on developmental change and clustering of observations within individuals over time, which we modeled using random effects at the individual level. Given the relatively small proportion of variance attributable to schools, adding a second clustering level would substantially increase model complexity and the risk of convergence problems, while offering

little substantive gain in precision. We therefore opted for more parsimonious two-level models (time within individuals). We have now clarified in the Methods section that (a) school-level clustering was examined via ICCs and (b) given the negligible school-level variance and the dissolution of clusters over time, we did not include school-level random effects in the main models (see p.9). Furthermore, we added to the limitation that “the slightly higher ICCs in young adulthood may indicate that early school and neighborhood environments contribute indirectly to the later stratification of generalized trust. We did not, however, include schools as a separate model level because, considering the small school-level ICCs and limited number of clusters, doing so would likely have introduced estimation challenges and substantially complicated the interpretation of SES and migration background effects.” (see p.20).

3. Using participants’ educational attainment at age 24 to predict trust measured at earlier ages may raise concerns regarding reverse causality or overcontrol bias. The authors should justify this modeling decision, clarify the theoretical rationale, or consider alternative specifications.

Response R1.4: We appreciate the reviewer’s thoughtful comment. We agree that including the highest educational attainment at age 24 alongside trust measured at earlier ages could raise concerns about reverse causality or overcontrol bias if interpreted causally. For this reason, education at 24 is not included in our main predictor models and is not used to adjust estimates of other predictors. Instead, we use it only in additional exploratory models as a distal life-course indicator to descriptively compare how generalized trust trajectories differ among youth who later follow diverging educational pathways. In these exploratory models, we substitute family SES with the highest achieved education, but do not draw conclusions about education causing earlier trust levels. To avoid overinterpretation, we now (a) explicitly label these analyses as exploratory and descriptive in the methods (see p.10) and in the results (see p.13), (b) deleted the figure displaying these findings alongside or main models, (c) clarified in the Discussion that they do not support causal inferences about education affecting earlier trust, and (d) emphasize that our main inferences about predictors of trust are based on models that do not condition on future educational attainment (see p.16).

4. Survey mode changes from paper-and-pencil to computer/online administration could influence observed mean-level differences. A brief discussion of potential mode effects would help contextualize the developmental trends.

Response R1.5: We agree that survey mode changes could, in principle, affect mean-level reports, particularly for sensitive behaviors. We have therefore added a short paragraph in the discussion noting this as a potential limitation. However, several large adolescent surveys that experimentally compared paper-and-pencil and electronic/computerized administration report largely comparable prevalence estimates for most outcomes, with only small mode effects for many indicators [1, 2]. Moreover, generalized trust is a relatively non-sensitive attitudinal construct (compared, for example, to substance use or other risk behaviors), for which mode-related underreporting or overreporting is less likely to be systematic. Taken together, while we cannot fully exclude mode effects, we consider it unlikely that the observed non-linear developmental pattern of generalized trust from ages 13 to 24 is substantially influeced by the shift from paper-and-pencil to computer-based/online surveys, and we now explicitly discuss this caveat in the manuscript. We have now added this to the limitation section (see p. 19, limitation 3).

5. Maternal trust is assessed using three binary items, which may limit reliability and reduce variability in estimates linking parental trust with adolescent trajectories. Discussing the reliability of this measure—and, more broadly, providing reliability estimates for all predictors—would enhance transparency.

Response R1.6: We thank the reviewer for this helpful suggestion. Maternal generalized trust was assessed with three dichotomous items adapted from the World Values Survey, and the resulting 0–3 total score showed acceptable internal consistency (Cronbach's α=0.62), given the small number of items and their intentionally broad coverage of different facets of trust. Reliability coefficients for maternal trust and all other sociodemographic, family-, and peer-level predictors (including time-invariant victimization) are reported in Supplementary Table 1, and we now more explicitly refer to this table in the Measures section to enhance transparency and mention that reliability estimates are to be found there (see p. 8-9).

Overall, this manuscript makes a meaningful contribution to understanding the development of generalized trust. Addressing these points might further enhance the interpretability of the findings.

Response R1.7: We thank the reviewer again for their time and thoughtful comments. We believe that addressing these points has further strengthened the manuscript and hope the reviewer will agree with this assessment.

Reviewer #2 (Remarks to the Author):
This manuscript describes a longitudinal study of the development of and the risk and protective factors of generalized trust from adolescence to young adulthood. The authors utilized longitudinal data collected from a large community sample of youth and their mothers who were living in Zurich, Switzerland, and incorporated a number of individual, interpersonal, and family-level risk and protective factors.

Response R2.1: We thank the reviewer for their time, thoughtful engagement with our work, and their productive, constructive review.

I appreciate the clear conceptualization of interpersonal trust and specifically generalized trust. However, it was odd to me to read the preceding paragraph on trust and child development (p. 3). To me, it would work better after the authors have provided the conceptual definition of trust.

Response R2.2: We appreciate this comment and understand that the current order may initially seem unusual. Our intention was to first provide a brief developmental framing of why trust matters for child and adolescent development, reflecting our primary perspective as developmental psychologists who examine trust through a developmental lens. In this opening subsection, we use the term trust in a deliberately broad sense to highlight its role in attachment, socioemotional development, resilience, and access to socio-ecological resources. We then narrow the focus in the subsequent section by formally defining generalized trust, distinguishing it from other forms of trust, and situating it within the specific trust literature, before finally reviewing the limited longitudinal work on generalized trust in adolescence and young adulthood. Because this structure mirrors our conceptual approach, from broad developmental relevance to a more specific construct definition and literature, we would prefer to retain the current order.

In the intro, the authors compared the cultural and experiential perspectives of trust (p. 4). I would like to see the authors link their findings back to the two perspectives again more explicitly in the discussion.

Response R2.3: We thank the reviewer for this thoughtful suggestion. In the revised Discussion, we now more explicitly link our findings back to cultural and experiential perspectives on generalized trust. Specifically, we added a subsection ("Cultural and Experiential Perspectives on Trust Development") in which we argue that the observed developmental pattern is best understood as the product of dynamic interactions between early socialization, structurally patterned cultural and socioeconomic embedding, and

proximal experiences across adolescence and young adulthood. In this integrative view, generalized trust is neither a purely fixed cultural trait nor merely a situational response, but reflects how early-formed expectations are progressively reinforced, modified, or attenuated through stratified and sometimes adverse experiences over the course of development (see on p. 17-18).

In most of the intro, the authors provided rationale for examining different risk and protective factors of generalized trust. Could the authors speak more about the importance of examining the developmental trajectory of generalized trust in and of itself?

Response R2.4: We appreciate this important point and have clarified the motivation for examining developmental trajectories more explicitly in the Introduction. Beyond identifying specific risk and protective factors, characterizing the shape and timing of generalized trust trajectories from adolescence into young adulthood is valuable in its own right because it reveals (a) when trust is most malleable versus increasingly stable, (b) whether changes are linear or non-linear, and (c) when social disparities begin to emerge or widen. This information is crucial for developmental theory and for pinpointing sensitive periods in which interventions or policy changes are most likely to have lasting effects on young people's trust in others and, by extension, on their mental health and societal participation. We have now added a sentence in the first paragraph (see p. 3), added a new header for the section on "The Developmental Course of Generalized Trust" and added two additional sentences emphasizing that describing mean-level and rank-order trajectories of generalized trust—is a core contribution, which then provides the developmental backbone for interpreting the effects of sociodemographic, family-, and peer-related predictors.

The authors reported random component of age ("minimal variance in age slopes…", p. 7). However, only linear age was allowed to randomly varying, but not quadratic age, is that correct?

Response R2.5: This is correct. In our baseline model fitting, we systematically compared alternative random-effects structures and found that including a random effect for quadratic age led to high collinearity with the random linear age term and convergence problems, without improving model fit (see Supplementary Methods 2, Supplementary Tables 2–3). We therefore retained random slopes only for the linear age term and a random intercept for ID, which provided stable estimation and captured individual differences in developmental change while avoiding overparameterization.

Why did the authors incorporate highest education attainment at age 24 as a predictor of trust in their final set of analysis?

Response R2.6: We agree that incorporating the highest educational attainment at age 24 alongside earlier measures of trust raises conceptual questions, so we now clarify that these models were designed as exploratory, outcome-oriented sensitivity analyses rather than as part of the main predictor framework. In this set of models, we substituted adolescent family SES with participants' eventual educational attainment and treated education at 24 as a distal life-course correlate that summarizes the educational pathways individuals followed, rather than as a causal determinant of earlier trust. As pointed out as well in our previous response (R1.4): To avoid overinterpretation, we now (a) explicitly label these analyses as exploratory and descriptive in the methods (see p.10) and in the results (see p.13), (b) deleted the figure displaying these results next to our main models, (c) clarify in the discussion that they do not support causal inferences about education affecting earlier trust, and (d) emphasize that our main inferences about predictors of trust are based on models that do not condition on future educational attainment (see p.16).

Did the authors recode the time variable age? If not, this would be a decision that does not make a lot of sense to me conceptually.

Response R2.7: We appreciate this opportunity to clarify our coding of time. In all longitudinal models, we recoded age so that the time variable reflects years since the baseline assessment (age 13), yielding values of 0, 2, 4, 7, and 11; thus, age 13 corresponds to time = 0, where the model intercept is located. This recoding places linear and quadratic age effects on a more interpretable scale and anchors change and curvature around the first measurement occasion. Chronological age in years is used only as the x-axis scale when visualizing trajectories to aid interpretability. To avoid confusion, we have moved the description of this recoding from the Supplementary Material to the main Methods section and explicitly highlighted the coding of time in the figure notes (see p. 9, p.24-26).

The way the analysis was described gives me the impression that, although there was a considerable amount of variability in the intercept (i.e., age 17 trust, given how age was coded), the authors did not use time-invariant predictors to explain between-person differences in the intercept. Rather, they used time-invariant predictors in their level-1 model (in hierarchical linear modeling terminology). If this was not what the authors did, I would like to see some LMM equations in the supplemental materials to clear the confusion. If indeed the authors did not examine between-person differences at baseline generalized trust, could the authors please explain their rationale for not doing so?

Response R2.7: We thank the reviewer for drawing attention to this point and agree that our original description may have been ambiguous.

Conceptually and statistically, we did use time-invariant predictors to explain between-person differences in the intercept; this intercept corresponds to generalized trust at age 13, because time was coded as years since baseline (0, 2, 4, 7, 11). All time-invariant predictors (e.g., sex, migration background, family SES, maternal trust, parenting measures) entered the model as fixed effects at the between-person level (Level 2) and thus account for variability in baseline trust and its developmental course via their interactions with age (Level 1), rather than being treated as time-varying predictors. For peer victimization, we additionally decomposed the time-varying measure into a between-person component (each person's mean level across waves) and a within-person component (wave-specific deviation from this mean), allowing us to model both stable differences and within-person fluctuations in victimization. The within-person peer victimization component was additionally used as a random slope.

To avoid further confusion, we have (a) clarified the coding of time and the age represented by the intercept in the Methods, and (b) added open code on OSF to the revision to show how intercepts and slopes are specified and where time-invariant and time-varying predictors enter the model, and (c) added code examples below for some of the models to clarify for the reviewer without having to seek these code snippets in the entire code files on OSF.

```
model_base <- lmer(GTrust_sc ~ age + I(age^2) + (1 + age | ID),
                   data = data_long, REML = FALSE,
                   control = lmerControl(optimizer = "bobyqa", optCtrl = list(maxfun = 1e5)))
```

Level 1 (within-person):

- Fixed effects of age and age2 model the average developmental trajectory of generalized trust over time.
- Age is a time-varying (Level-1) predictor that captures within-person change in trust across measurement occasions.

Level 2 (between-person):

- **The term (1+age|ID) includes a random intercept for each ID, representing person-specific baseline deviations from the grand mean at age = 0, and a random slope for the Level-1 age predictor, allowing the within-person age effect to vary across individuals (i.e., each person has their own linear change over time).**

Together, the random intercept and random age slope define a person-specific growth curve around the population-average fixed trajectory.

```
model_soc<- lmer(GTrust_sc ~  age + I(age^2) + sex + K456_ISEImax + MigHH2 +
                                        (1 + age| ID),
                data = data_long, REML=FALSE,
                control = lmerControl(optimizer = "bobyqa", optCtrl = list(maxfun = 1e5)))
```

Level 2 (between-person):
- sex, K456_ISEImax (family SES), MigHH2 (migration background) are time-invariant person-level predictors.
- They enter as fixed effects on the random intercept and linear age slope in the sense that they shift the average level of trust (baseline, age = 0) and, because age is also in the model, the group-specific trajectory is the sum of intercept + age terms + these main effects. In this specification, they do not explicitly change the shape of the growth curve over time; they shift the whole curve up or down.

```
model_socInt<- lmer(GTrust_sc ~ (age + I(age^2)) * (sex + K456_ISEImax + MigHH2) +
                                                (1 + age| ID),
                data = data_long, REML=FALSE,
                control = lmerControl(optimizer = "bobyqa", optCtrl = list(maxfun = 1e5)))
```

Level 1 (Age) * Level 2 (between-person): the sociodemographic variables now moderate both the linear and quadratic time effects, i.e., they explain between-person differences in:
- baseline level (via their main effects), and
- the shape of growth trajectories (via interactions with age and age^2).

```
model_vic <- lmer(GTrust_sc ~ (bullying_between + bullying_within) + age + I(age^2) +
                        P2_trust_new + div13 + aversive13 + inv13c) + sex + K456_ISEImax +
                        MigHH2+ 1 + age + bullying_within| ID),
                data = data_long, REML = FALSE,
                control = lmerControl(optimizer = "bobyqa", optCtrl = list(maxfun = 1e5)))

model_vicint <- lmer(GTrust_sc ~  (bullying_between + bullying_within) * (age  + I(age^2)) +
                        (P2_trust_new + div13 + aversive13 + inv13c) + sex + K456_ISEImax
                        + MigHH2 + (1 + age + bullying_within| ID),
                data = data_long, REML = FALSE,
                control = lmerControl(optimizer = "bobyqa", optCtrl = list(maxfun = 1e5)))
```

To examine whether the effects of peer victimization depend on developmental timing (aim 4), we included interactions of both the between-person and within-person victimization components with linear and quadratic age. This specification allows victimization to moderate not only the average level of generalized trust, but also the shape of trust trajectories across adolescence and young adulthood.

In this model, bullying_between × age and bullying_between × age^2 test whether youths who are chronically more victimized than their peers show different linear or non-linear changes in generalized trust over time. Likewise, bullying_within × age and bullying_within × age^2 test whether the short-term impact of deviations from a youth’s typical victimization level on generalized trust varies across developmental stages. To test whether the random slope for

bullying_within improves model fit, we compared the model versions with and without this random slope against each other.

We hope these explanations, together with the changes to the statistical analyses section, clarify the confusion, which we believe was primarily about terminology rather than about the underlying modeling strategy.

Please provide more details about how the analytic sample was determined.

Response R2.8: We have clarified the construction of the analytic sample in the revised manuscript. All cohort members who participated in at least one trust assessment of the five focal waves (ages 13, 15, 17, 20, or 24) and who provided at least one valid generalized trust score were included, yielding an analytic sample of 1,481 adolescents and young adults (see corresponding changes on p. 7 & 9). Because longitudinal linear mixed models can accommodate missing outcomes under missing-at-random assumptions, participants were not excluded if they missed one or more later waves; instead, all available trust observations contributed to the estimation of growth curves. In contrast, missing data on predictor variables were addressed using multiple imputation by chained equations, with an extensive set of sociodemographic, family, and auxiliary variables to model missingness, which is now described in Supplementary Methods 1 and explicitly linked in the Methods section.

Also, did the authors conduct any longitudinal attrition analysis?

Response R2.9: We did not run a separate, dedicated longitudinal attrition analysis for this paper, but we relied on and cited existing attrition work from the z-proso cohort and explicitly addressed missingness in our imputation strategy. In Supplementary Methods 1, we describe patterns of missing data, show that 41.1% of participants had complete data and 12.5% of all data points were missing, and explain that we assumed missing at random conditional on well-documented predictors of non-response and attrition in z-proso (e.g., sociodemographic factors, earlier bullying). These variables, along with auxiliary indicators related to participation and key study constructs, were included in the imputation models to reduce potential attrition bias.

Reviewer #3 (Remarks to the Author):
This is an excellent and well-written study on a very impressive dataset. The analyses are well conducted and the conclusions are well supported by the data. I have a couple of suggestions that are hopefully useful for the authors when revising the manuscript.

Response R3.1: We thank the reviewer for their positive evaluation of our study and for highlighting the strengths of the dataset, analyses, and conclusions. We appreciate their constructive suggestions and have carefully considered and incorporated them in the revised manuscript where possible, noting specific changes in the point-by-point responses below.

First and most important, I have some doubts whether the 3-item questionnaire actually measures generalized trust. Questions are: ‘most people can be trusted’, ‘people usually try to help other people’, ‘most people try to be fair’. A declining score between ages 13-17 is interpreted as that adolescents become less trusting, but one could also argue that they become more realistic. We teach our adolescents that not everyone can be trusted (e.g. online, but also in real life). The increase again between ages 17-24 can also be related to more selective environments where individuals are actually more trustworthy. This is important for the framing of the study where it is assumed that more trust is always better.

Response R3.2: We appreciate this thoughtful comment and agree that both the content of our three items and the developmental context invite a nuanced interpretation. The items we

used (“most people can be trusted”, “people usually try to help other people”, “most people try to be fair”) are a widely used proxy for generalized trust in large-scale surveys (e.g., World Values Survey) and have been employed in select prior developmental work on adolescents’ trust beliefs, which allows comparability across studies and cohorts. At the same time, we fully agree that a decline between ages 13 and 17 can also reflect adolescents’ increasingly realistic calibration of others’ trustworthiness (e.g., through online experiences and broader social exposure), and that a later increase may partly mirror entry into more selective, trustworthy environments rather than a simple “return” to naïve trust.

In response, we have revised the sections of the discussion to (a) emphasize that higher generalized trust is not uniformly optimal in every context, (b) explicitly acknowledge that developing a more discerning, context-sensitive trust stance can be adaptive, and (c) highlight that our findings speak to changes in adolescents’ broad expectations about “most people,” which likely combine both increased realism and shifts in actual social environments. We now also clarify that we interpret the observed trajectory as reflecting both changes in generalized trust and developmental recalibration of social expectations, rather than assuming that “more trust is always better.” Please see changes on p. 15 & 20.

Relatedly, the authors argue that there is no prior research on trust in adolescents, but there is actually a very large literature on these topics with more extensive trust-measuring questionnaires and trust games. I am not saying that this makes the current study less important: I believe these are data that should be published, but the authors could be more balanced with referring to prior developmental literature and the specific contribution of this study.

Response R3.3: We thank the reviewer for pointing out that there is a larger developmental literature on trust in adolescence that uses more extensive questionnaires, trust games, and other operationalizations of trust. In line with our responses to the other reviewers, we have now more carefully distinguished between the relatively small body of work on the development of generalized trust and trust beliefs in adolescence and young adulthood and the comparably larger literature on broader trust conceptualizations, including behavioral trust games, particularized trust toward specific targets, and non-interpersonal or institutional trust. We also now explicitly discuss in the Introduction and Discussion that different trust forms and measures often show distinct developmental trajectories across adolescence—for example, increasing or stable behavioral trust in trust games versus U-shaped or declining self-reported generalized trust-which underscores that these constructs are related but not interchangeable (p.4-5, 15, 20). We hope that these revisions provide a more balanced positioning of our study within the broader developmental trust literature, while clarifying that our contribution is specifically to the longitudinal development of generalized trust beliefs rather than to all forms of adolescent trust.

Specifically, we revised the introduction and more specifically elaborated on the gap in the context of the broader trust literature:
“Although there is a growing empirical literature on trust in childhood and adolescence, including studies using more extensive questionnaires and behavioral trust games, this work is dispersed across disciplines and often uses diverse labels for related constructs, which makes it difficult to integrate into a coherent developmental picture. In particular, longitudinal research specifically on generalized trust beliefs over time remains relatively sparse.” (see p. 4)

Moreover, we believe that discussing more specifically distinct forms of trust in limitation point 1, highlights the specific contribution to this particular form and measure of trust and not perse to the larger trust literature and different forms of trust being measured differently. See highlighted sections on p. 20.

Finally, in the discussion the authors suggest that the solution for more generalized trust lies in civic engagement. But most of their results show that drops in generalized trust are related to societal encounters, so shouldn't the solution be also that our societies should provide more opportunities to trust others. This can possibly added for a balanced interpretation.

Response R3.4: We thank the reviewer for this important point. In the revised implication section, we now note more concisely that social engagements in adolescence can function both as sources of harm (e.g., exclusion, victimization) and as opportunities to experience others as fair and reliable. We therefore complement our earlier focus on civic engagement by emphasizing the need for social contexts that maximize positive, cooperative interactions while minimizing harmful experiences, so that young people can build generalized trust through "as harm-free as possible" everyday engagement with others. Please see changes on p. 21.

References

1. Colasante, E., E. Benedetti, L. Fortunato, M. Scalese, R. Potente, A. Cutilli, and S. Molinaro, *Paper-and-pencil versus computerized administration mode: Comparison of data quality and risk behavior prevalence estimates in the European school Survey Project on Alcohol and other Drugs (ESPAD).* PLoS One, 2019. **14**(11): p. e0225140.https://doi.org/10.1371/journal.pone.0225140.
2. Lucia, S., L. Herrmann, and M. Killias, *How important are interview methods and questionnaire designs in research on self-reported juvenile delinquency? An experimental comparison of Internet vs paper-and-pencil questionnaires and different definitions of the reference period.* Journal of Experimental Criminology, 2007. **3**(1): p. 39-64.https://doi.org/10.1007/s11292-007-9025-1.
