# Supplementary material for: Family background and peer victimization are associated with generalized trust from adolescence to adulthood
Source: Commun Psychol. 2026 May 22;4:116. doi: 10.1038/s44271-026-00474-z (PMC13473010; doi:10.1038/s44271-026-00474-z)
Supplement: Supplementary file 2 [file 44271_2026_474_MOESM2_ESM.pdf]

Supplementary Material:

Family background and peer victimization are associated with generalized trust from adolescence to adulthood

Index

Supplementary Table 1. Measures description of sociodemographic, family-level and peer-level predictors of generalized trust. 2

Supplementary Figure 1. Pearson Correlation Matrix and corresponding significance levels of all study variables. 5

Supplementary Figure 2. Display of missing data patterns. 6

Supplementary Methods 1: Description of Multiple Imputation Procedure. 7

Supplementary Methods 2: Description of Baseline Model Fitting: Model Structure and Fixed and Random Effects of Age and Age^2 7

Supplementary Table 2. Estimates, confidence intervals, and fit statistics of baseline model versions predicting generalized trust. 8

Supplementary Table 3. Model comparison of baseline models with different specification. 9

Supplementary Table 4. Sociodemographic and family-level predictors of generalized trust. 10

Supplementary Table 5. Exploratory analyses for highest educational attainment. 12

Supplementary Table 6. Model comparison of two nested models with and without a random slope for bullying-within. 14

Supplementary Table 7. Between- and within-person bullying predicting generalized trust. 15

References 16

Supplementary Table 1. Measures description of sociodemographic, family-level and peer-level predictors of generalized trust.

Construct	Measures description	Cronbach's α	Scale/Coding
**Sociodemographic factors (time-invariant, single measurements)**			
**Sex**	Child sex assigned at birth was available from the official school records [1].	--	Binary variable (0=male; 1=female)
**Migration background**	Migration background was assessed using parental country of birth reported by focal participants. A composite score was created based on information from when participants were 11, 13, and 15 to maximize information. [1]	--	Binary variable (0= at least one parent born in Switzerland; 1= both parents born abroad)
**Family socioeconomic status (SES)**	Family SES was assessed using the International Socio-Economic Index of Occupational Status [2] based on parental occupation reported by focal participants. This score captures occupation-specific income and required educational level. We used the highest ISEI score based on information from ages 11, 13, and 15 to maximize information [1].	--	Dimensional: 16 (e.g., unskilled worker) to 90 (e.g., judge). Standardized scores (z-scaled) were entered into models.
**Highest educational attainment**	We assessed participants highest educational attainment at the most recent assessment wave at age 24 by self-report. From initial detailed responses based on specifics of the Swiss educational system, six categories were coded [1]. Lower than apprenticeship was used as reference category in statistical models and dummy coded items corresponding to the other five categories were entered into the models.	-	Categorical variable: below apprenticeship, apprenticeship vocational baccalaureate, baccalaureate, vocational tertiary academic tertiary. Dummy coded variables in models, ref. category: below apprenticeship.

Family-level factors (time-invariant, single measurements)			
**Maternal generalized trust**	Maternal generalized trust was measured with three items adapted from the World Value Survey [3, 4]. The items asked: (1) “Generally speaking, would you say that most people can be trusted or that you can’t be too careful in dealing with people?”, (2) “Would you say that most of the time the people that you come into contact with try to be helpful or are they mostly looking out for themselves?”, and (3) “Do you think that most people that you come into contact with would try to take advantage of you if they got the chance or would they try to be fair?”. For each participant, scores on these three items were added together, and then 3 was subtracted from the sum to create a total score. Higher total scores indicate greater overall trust.	0.62	Binary scale. *Item 1:* 1 = Most people can be trusted, 2 = Need to be very careful *Item 2:* 1 = Most people try to be helpful, 2 = Most people look out for themselves. *Item 3:* 1 = Would take advantage, 2 = Would try to be fair (reverse coded). Total score ranging from 0 (lowest trust) to 3 (highest trust).
**Parental involvement**	Parental involvement was measured at the age 13 assessment. Participant-rated on 6 items (e.g., “You play games or do fun things with your parents”) of the Alabama Parenting Questionnaire (APQ) [5]. These items reflected various aspects of positive parent–child engagement, including talking with the child, providing comfort, showing interest, being approachable for problems, engaging in play or activities together, and helping with homework.	0.75	Mean score: four-point Likert scale from “never” to “often”. Standardized scores (z-scaled) were entered into models.
**Parental separation /divorce**	Parental separation/divorce was reported by focal participants at age 13.	--	Binary variable (0= not separated/divorced; 1= separated/divorced)
**Aversive parenting**	Participants reported exposure to aversive parenting at age 13. The 5 items from the APQ [5]. These included instances where parents reportedly slapped, spanked, pulled the child's ears or hair, became more severe in their discipline when in a bad mood, or yelled at the participant. Each item reflects a specific aspect of harsh disciplinary practices within the family context.	0.72	Mean score: four-point Likert scale from “never” to “always”. Standardized scores (z-scaled) were entered into models.

Peer-level: Bullying victimization (time-variant, longitudinal measurements)			
**Bullying victimization**	Bullying victimization was assessed using 5 items of the Zurich Bullying Scale [6, 7]. These items reflected the experiences of peer victimization in the last 12 months. Items asked if participants were 'purposely ignored or excluded', 'mocked, or insulted', 'hit, bitten or kicked or hair-pulled', if others 'took or destroyed belonging', or others 'sexually harassed'. Items were framed to refer to other adolescents (assessments at age 13, 15, 17) and others (assessments at age 20, 24) including peers and others from any social context. The context at assessments ages 13, 15, was further specified as: 'for example, at school, on the way to school, when being out, at home, or on the internet'. At ages 17, 20, 24, 'at the workplace' was added to that list.	Age 13=0.70[1]<br>Age 15=0.64<br>Age 17=0.62<br>Age 20=0.60<br>Age 24=0.57[2]	Mean score: six-point Likert scale: 1= never, 2= 1-2 times, 3=3-10 times, 4= approximately once a month, 5= approximately once a week, and 6= (almost) daily. Standardized scores (z-scaled) were entered into models.

[1] The scale intentionally covers different forms of bullying (social exclusion, verbal, physical, sexual), making the items less homogeneous. Items that tap distinct experiences reduce internal consistency.

[2] At age 24 only 4 items were used to build the scale, as one item (took or destroyed belongings) was no longer included.

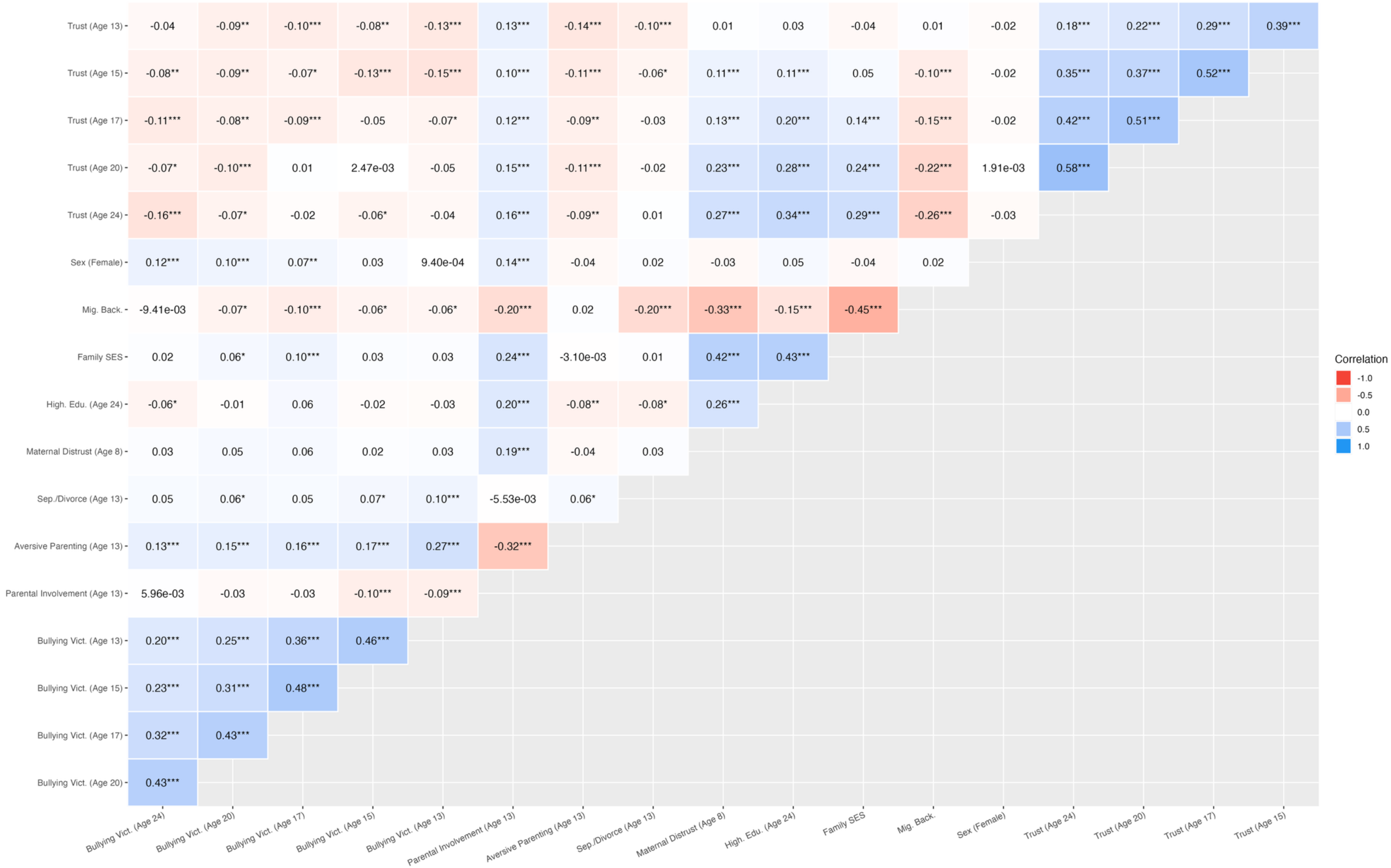

Supplementary Figure 1. Pearson Correlation Matrix and corresponding significance levels of all study variables.

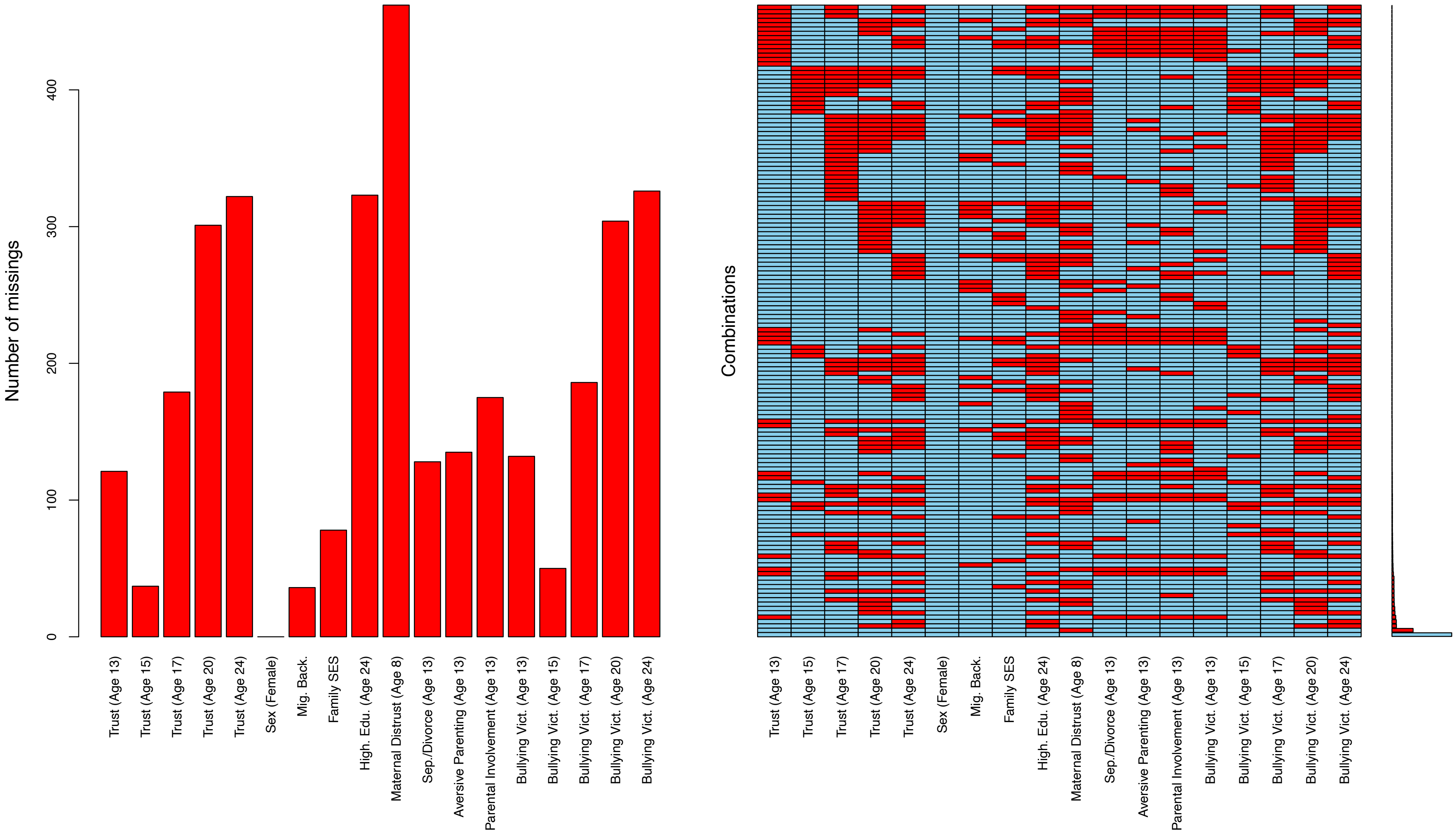

Supplementary Figure 2. Display of missing data patterns.

Left: Number of missings per item for all study variables of interest; *Right:* Combinations of missings across variables. Overall, 41.4% of participants had complete data in all study variables and 12.5% of all datapoints were missing.

Supplementary Methods 1: Description of Multiple Imputation Procedure.

We investigated patterns of missingness for all included variables (see patterns of missingness displayed in Supplementary Figure 2). Overall, 41.4% of participants had complete data in all study variables, and 12.4% of all data points were missing. Under the assumption of missingness at random (MAR) with well-known factors contributing to missingness/attrition in the z-proso cohort [8]. We specified different types of imputation models depending on the variable types to be imputed: using predictive mean matching for dimensional variables, logistic regressions for binary variables, and polynomial regressions for multicategorical variables in MICE [9]. MICE was chosen because it preserves the original distribution of the data and is robust to non-normality. We predicted missing variables only in predictor variables, not trust data, as linear mixed models (LMMs) can accommodate partial missingness within outcomes. However, within the imputation models, trust was used as predictor variables to impute missing data in other variables. Additionally, we included other auxiliary items into the missingness prediction models as these are available most participants and are related to our study variables (items: religious affiliation, class type at age 13, detailed migration background, and bullying victimization at age 8) [1].

Supplementary Methods 2: Description of Baseline Model Fitting: Model Structure and Fixed and Random Effects of Age and Age^2

To account for the repeated measures structure of the data, participant ID was modeled as a random intercept, thereby adjusting for inter-individual differences and the non-independence of observations within participants. We first compared several baseline models for optimal model specification. Baseline models included (1) trust as outcome and only a random intercept, (2) a fixed linear age term and a random intercept, (3) a fixed linear and quadratic age term and a random intercept, (4) a fixed linear and quadratic age term and a random slope for linear age and a random intercept. A model including both fixed and random effects for linear and quadratic age did not converge. The model was nearly unidentifiable with very large eigenvalue, indicating overparameterization and resulting multicollinearity in the random effects structure. The estimates, confidence intervals, and fit statistics from these models are summarized in Supplementary Table 2. We used the *compare_performance* function from the *performance*-package to test models against each other [10]. The best fitting model was model 4 (see Supplementary Table 2 and 3).

Supplementary Table 2. Estimates, confidence intervals, and fit statistics of baseline model versions predicting generalized trust.

	Baseline Model (Model 1)			Linear Model (Model 2)			Linear Model with Random Age Slope (Model 3)			Quadratic Model with Random Age Slope (Model 4)		
Predictors	*Estimates*	*95%-CI*	*p*	*Estimates*	*95%-CI*	*p*	*Estimates*	*95%-CI*	*p*	*Estimates*	*95%-CI*	*p*
(Intercept)	-0.00	[-0.04; 0.04]	0.969	0.11	[0.07; 0.16]	**<0.001**	0.12	[0.07; 0.16]	**<0.001**	0.26	[0.21; 0.31]	**<0.001**
Age				-0.03	[-0.03; -0.02]	**<0.001**	-0.03	[-0.03; -0.02]	**<0.001**	-0.14	[-0.15; -0.12]	**<0.001**
Age^2										0.01	[0.01; 0.01]	**<0.001**
**Random Effects**												
σ^2	0.64			0.62			0.53			0.51		
τ_{00}	0.36_{ID}			0.36_{ID}			0.37_{ID}			0.38_{ID}		
τ_{11}							$0.01_{ID.age}$			$0.01_{ID.age}$		
ρ_{01}							-0.25_{ID}			-0.26_{ID}		
ICC	0.36			0.37			0.46			0.48		
N	1481_{ID}			1481_{ID}			1481_{ID}			1481_{ID}		
Observations	6445			6445			6445			6445		
Marginal & Cond.R^2	0.000 / 0.363			0.010 / 0.374			0.011 / 0.470			0.026 / 0.493		

Note. CI=Confidence Interval. σ^2: Residual variance; unexplained within-person variation.
τ_{00}: Random intercept variance (ID); baseline differences between participants.
τ_{11}: Random slope variance (ID.age); individual differences in age trends. ρ_{01}: Correlation of random intercepts and slopes; link between baseline trust and age effect. ICC: Intraclass correlation; proportion of total variance between participants. N: Number of unique participants (IDs). Observations: Total data points (all waves and participants).
Marginal R^2 / Conditional R^2: Variance explained by fixed effects / by full model (fixed & random).

Supplementary Table 3. Model comparison of baseline models with different specification.

Model	**Conditional R^2**	**Marginal R_2**	**ICC**	**RMSE**	**Sigma**	**Performance Score**
**Model 4** (fixed age, age^2, random slope age and random intercept)	0.4926	0.0256	0.4792	0.3884	0.4495	1.0000
**Model 3** (fixed age, age^2, and random intercept)	0.4702	0.0111	0.4642	0.3990	0.4596	0.4743
**Model 2** (fixed age and random intercept)	0.3745	0.0096	0.3684	0.4559	0.4980	0.0818
**Model 1** (Intercept only)	0.3632	0.0000	0.3632	0.4604	0.5026	0.0000

Note. Conditional R^2: Variance explained by the full model (fixed and random effects). Marginal R^2: Variance explained by fixed effects only. ICC: Proportion of variance due to between-participant differences. RMSE: Root mean square error; average model prediction error. Sigma: Residual standard deviation. Performance Score: Relative fit index (best model = 1.00) from the performance package in R [10].

Supplementary Table 4. Sociodemographic and family-level predictors of generalized trust.

	Sociodemographic (Model 1)			Sociodemographic x Age and Age^2 (Model 2)			Family-level, Sociodemographic (Model 3)			Family-level x Age and Age^2 Sociodemographic (Model 4)		
Predictors	*Estimates*	*95%-CI*	*p*	*Estimates*	*95%-CI*	*p*	*Estimates*	*95%-CI*	*p*	*Estimates*	*95%-CI*	*p*
(Intercept)	0.36	[0.29; 0.43]	**<0.001**	0.29	[0.21; 0.38]	**<0.001**	0.39	[0.32; 0.48]	**<0.001**	0.41	[0.33; 0.49]	**<0.001**
**Age**	-0.14	[-0.15; -0.12]	**<0.001**	-0.11	[-0.14; -0.08]	**<0.001**	-0.14	[-0.15; -0.12]	**<0.001**	-0.14	[-0.16; -0.12]	**<0.001**
**Age^2**	0.01	[0.01; 0.01]	**<0.001**	0.01	[0.01; 0.01]	**<0.001**	0.01	[0.01; 0.01]	**<0.001**	0.01	[0.01; 0.01]	**<0.001**
**Sex**	-0.02	[-0.09; 0.05]	0.540	-0.04	[-0.14; 0.05]	0.358	-0.04	[-0.11; 0.03]	0.273	-0.04	[-0.11; 0.03]	0.287
**Family SES**	0.07	[0.03; 0.11]	**0.001**	-0.06	[-0.11; 0.00]	**0.035**	0.02	[-0.02; 0.07]	0.265	0.02	[-0.02; 0.07]	0.277
**Migration Background**	-0.17	[-0.25; -0.09]	**<0.001**	-0.03	[-0.13; 0.08]	0.634	-0.15	[-0.24; -0.07]	**<0.001**	-0.15	[-0.24; -0.07]	**<0.001**
Age × Sex				0.01	[-0.02; 0.05]	0.421						
Age × Fam. SES				0.04	[0.03; 0.06]	**<0.001**						
Age × Mig. Back.				-0.07	[-0.11; -0.03]	**<0.001**						
Age^2× Sex				-0.00	[0.00; 0.00]	0.448						
Age^2× Fam. SES				-0.00	[0.00; -0.00]	**0.044**						
Age^2× Mig. Back.				0.00	[0.00; 0.01]	**0.017**						
**Maternal Trust**							0.08	[0.07; 0.16]	**0.001**	-0.03	[-0.09; 0.03]	0.301
**Divorce/Separation**							-0.12	[-0.19; -0.03]	**0.004**	-0.21	[-0.32; -0.10]	**<0.001**
**Aversive Parenting**							-0.08	[-0.13; -0.05]	**<0.001**	-0.10	[-0.15; -0.05]	**<0.001**
**Parental Involvement**							0.06	[0.02; 0.10]	**0.005**	0.06	[0.00; 0.11]	**0.039**
Age × Maternal Trust										0.04	[0.02; 0.06]	< .001

Age × Divorce/Separation										0.03	[-0.01; 0.07]	0.103
Age × Aversive Par.										0.01	[-0.01; 0.03]	0.292
Age × Par. Involvement										-0.00	[-0.02; 0.02]	0.823
Age^2 × Maternal Trust										-0.00	[0.00; 0.00]	0.104
Age^2 × Divorce/Separation										-0.00	[0.00; 0.00]	0.422
Age^2 × Aversive Parenting										-0.00	[0.00; 0.00]	0.440
Age^2 × Par. Involvement										0.00	[0.00; 0.00]	0.640
**Random Effects**												
σ^2	0.51			0.51			0.51			0.51		
τ_{00}	0.40 $_{ID}$			0.38 $_{ID}$			0.38 $_{ID}$			0.36 $_{ID}$		
τ_{11}	0.01 $_{ID.age}$			0.00 $_{ID.age}$			0.01 $_{ID.age}$			0.00 $_{ID.age}$		
ρ_{01}	-0.35 $_{ID}$			-0.30 $_{ID}$			-0.37 $_{ID}$			-0.32 $_{ID}$		
ICC	0.47			0.46			0.45			0.44		
N	1481 $_{ID}$			1481 $_{ID}$			1481 $_{ID}$			1481 $_{ID}$		
Observations	6445			6445			6445			6445		
Marginal R^2 / Conditional R^2	0.043 / 0.488			0.068 / 0.494			0.069 / 0.488			0.087 / 0.490		

Note. CI = Confidence Interval. σ^2: Residual variance; unexplained within-person variation.
τ_{00}: Random intercept variance (ID); baseline differences between participants.
τ_{11}: Random slope variance (ID.age); individual differences in age trends.
ρ_{01}: Correlation of random intercepts and slopes; link between baseline trust and age effect.
ICC: Intraclass correlation; proportion of total variance between participants.
N: Number of unique participants (IDs). Observations: Total data points (all waves and participants).
Marginal R^2 / Conditional R^2: Variance explained by fixed effects / by full model (fixed & random).

Supplementary Table 5. Exploratory analyses for highest educational attainment.

	**Sociodemographic (highest educ. Attainm.) (Model 1b)**			**Sociodemographic (highest educ. Attainm.) x Age and Age^2 (Model 2b)**		
Predictors	*Estimates*	*95%-CI*	*p*	*Estimates*	*95%-CI*	*p*
(Intercept)	0.26	[0.12; 0.39]	**<0.001**	0.31	[0.13; 0.49]	< .001
**Age**	-0.14	[-0.15; -0.12]	**<0.001**	-0.15	[-0.21; -0.08]	< .001
**Age^2**	0.01	[0.01; 0.01]	**<0.001**	0.01	[0.00; 0.01]	0.002
**Sex**	-0.04	[-0.11; 0.03]	0.248	-0.04	[-0.13; 0.06]	0.437
**Edu. Attain. Ref. [below apprenticeship]**						
**Edu. [apprenticeship]**	0.02	[-0.12; 0.16]	0.796	-0.02	[-0.20; 0.17]	0.853
**Edu. [vocational baccalaureate]**	0.09	[-0.07; 0.25]	0.285	-0.08	[-0.29; 0.13]	0.483
**Edu. [baccalaureate]**	0.22	[0.06; 0.39]	**0.006**	-0.18	[-0.41; 0.04]	0.102
**Edu. [vocational tertiary]**	0.16	[0.00; 0.33]	0.054	-0.00	[-0.23.; 0.22]	0.966
**Edu. [academic tertiary]**	0.47	[0.31; 0.63]	**<0.001**	0.04	[-0.18; 0.26]	0.717
**Mig.**	-0.19	[-0.27; -0.12]	**<0.001**	0.02	[-0.08; 0.11]	0.751
Age × Sex				0.01	[-0.03; 0.04]	0.740
Age × Edu. Attain. Ref. [below apprenticeship]				0.01	[-0.06; 0.08]	0.781
Age × Edu. apprenticeship				0.03	[-0.05; 0.10]	0.484
Age × Edu. vocational baccalaureate				0.15	[0.07; 0.23]	< .001
Age × Edu. baccalaureate				0.05	[-0.04; 0.13]	0.264
Age × Edu. vocational tertiary				0.14	[0.06; 0.22]	< .001
Age × Mig				-0.09	[-0.12; -0.05]	< .001
Age^2 × Sex				-0.00	[0.00; 0.00]	0.558
Age^2 × *Edu. apprenticeship*				-0.00	[-0.01; 0.01]	0.997
Age^2 × *Edu. vocational baccalaureate*				0.00	[0.00; 0.01]	0.522
Age^2 × *Edu. baccalaureate*				-0.00	[-0.01; 0.00]	0.056

Age^2 × *Edu. vocational tertiary*				-0.00	[-0.01; 0.01]	0.913
Age^2 × *Edu. academic tertiary*				-0.00	[-0.01; 0.00]	0.208
Age^2× Sex				0.00	[0.00; 0.01]	0.002
**Random Effects**						
σ^2	0.51			0.50		
τ_{00}	0.40 $_{ID}$			0.38 $_{ID}$		
τ_{11}	0.01 $_{ID.age}$			0.00 $_{ID.age}$		
ρ_{01}	-0.41 $_{ID}$			-0.36 $_{ID}$		
ICC	0.45			0.44		
N	1481 $_{ID}$			1481 $_{ID}$		
Observations	6445			6445		
Marginal R^2 / Conditional R^2	0.064 / 0.487			0.097 / 0.494		

Note. CI = Confidence Interval. σ^2: Residual variance; unexplained within-person variation.
τ_{00}: Random intercept variance (ID); baseline differences between participants.
τ_{11}: Random slope variance (ID.age); individual differences in age trends.
ρ_{01}: Correlation of random intercepts and slopes; link between baseline trust and age effect.
ICC: Intraclass correlation; proportion of total variance between participants.
N: Number of unique participants (IDs). Observations: Total data points (all waves and participants).
Marginal R^2 / Conditional R^2: Variance explained by fixed effects / by full model (fixed & random).

Supplementary Table 6. Model comparison of two nested models with and without a random slope for bullying-within.

**Metric**	**Model 5 (see below)**	**Model 5 (without random slope for bullying within)**
AIC	16571.2	16576.7
AIC Weight	0.941	0.059
BIC	16699.8	16685.1
BIC Weight	0.00062	0.999
R^2 (conditional)	0.502	0.490
R^2 (marginal)	0.081	0.081
ICC	0.458	0.445
RMSE	0.604	0.617
Sigma	0.703	0.711
Performance Score	**87.50%**	12.50%

Note. AIC: Model selection criterion balancing fit and complexity; lower values indicate better models. BIC: Like AIC, but applies a stronger penalty for model complexity; favors simpler models with large samples. AIC/BIC weights: The probability that a model is the best among those compared, given the data. Conditional R^2: Variance explained by the full model (fixed and random effects). Marginal R^2: Variance explained by fixed effects only. ICC: Proportion of variance due to between-participant differences. RMSE: Root mean square error; average model prediction error. Sigma: Residual standard deviation. Performance Score: Relative fit index (best model = 1.00) from the performance package in R [10].

Supplementary Table 7. Between- and within-person bullying predicting generalized trust.

	Bullying on Trust (Model 5)			Bullying x Age and Age² on Trust (Model 6)		
Predictors	*Estimates*	*95%-CI*	*p*	*Estimates*	*95%-CI*	*p*
(Intercept)	0.42	[0.35; 0.50]	**<0.001**	0.40	[0.33; 0.48]	**<0.001**
**Bullying (between)**	-0.14	[-0.19; -0.08]	**<0.001**	-0.17	[-0.25; -0.10]	**<0.001**
**Bullying (within)**	-0.08	[-0.11; -0.06]	**<0.001**	0.00	[-0.05; 0.05]	0.975
Age	-0.14	[-0.16; -0.13]	**<0.001**	-0.14	[-0.16; -0.12]	**<0.001**
Age^2	0.01	[0.01; 0.01]	**<0.001**	0.01	[0.01; 0.01]	**<0.001**
***Age x* Bullying (between)**				0.01	[-0.01; 0.04]	0.260
***Age² x* Bullying (between)**				-0.00	[0.00; 0.00]	0.306
***Age x* Bullying (within)**				-0.04	[-0.07; -0.01]	**0.002**
***Age² x* Bullying (within)**				0.00	[0.00; 0.01]	**0.034**
Sex	-0.02	[-0.09; 0.05]	0.536	-0.02	[-0.09; 0.05]	0.631
Fam. SES	0.03	[-0.01; 0.07]	0.175	0.03	[-0.01; 0.07]	0.153
Mig.	-0.17	[-0.25; -0.08]	**<0.001**	-0.17	[-0.25; -0.08]	**<0.001**
Mat. Trust	0.08	[0.03; 0.12]	**<0.001**	0.08	[0.03; 0.12]	**<0.001**
Divorce	-0.11	[-0.19; -0.02]	**0.011**	-0.11	[-0.19; -0.03]	**0.010**
Aversive Par.	-0.06	[-0.10; -0.02]	**0.004**	-0.06	[-0.10; -0.02]	**0.003**
Par. Involvement	0.05	[0.01; 0.10]	**0.008**	0.05	[0.01; 0.10]	**0.008**
**Random Effects**						
σ^2	0.49			0.49		
τ_{00}	0.38_{ID}			0.38_{ID}		
τ_{11}	$0.01_{ID.age}$			$0.01_{ID.age}$		
	$0.02_{ID.bullying\_within}$			$0.02_{ID.bullying\_within}$		
ρ_{01}	-0.38			-0.38		
	-0.17			-0.17		
ICC	0.46			0.46		
Observations	6445			6445		
Marginal R^2 / Conditional R^2 R2 / R2 adjusted	0.081 / 0.502			0.084 / 0.504		

Note. 95%-CI=95% Confidence Interval. σ^2: Residual variance; unexplained within-person variation.
τ_{00}: Random intercept variance (ID); baseline differences between participants.
τ_{11}: Random slope variance (ID.age) and (ID.bullying_within); individual differences in age trends and in sensitivity to bullying.
ρ_{01}: Correlation of random intercepts and slopes; link between baseline trust and age effect.
ICC: Intraclass correlation; proportion of total variance between participants.
N: Number of unique participants (IDs). Observations: Total data points (all waves and participants).
Marginal R^2 / Conditional R^2: Variance explained by fixed effects / by full model (fixed & random).

R2 / R2 adjusted: Proportion of variance in the outcome explained by all model predictors/ R^2 corrected for the number of predictors; accounts for potential model overfitting.

References

1. z-proso Project Team, *z-proso Handbook: Instruments and Procedures in the Adolescent and Young Adult Surveys (Age 11 to 20; Waves K4-K9). Short Version.* 2024.https://doi.org/10.5167/uzh-257984.
2. Ganzeboom, H.B., P.M. De Graaf, and D.J. Treiman, *A standard international socio-economic index of occupational status.* Social science research, 1992. **21**(1): p. 1-56,
3. Delhey, J., K. Newton, and C. Welzel, *How General Is Trust in "Most People"? Solving the Radius of Trust Problem.* American sociological review, 2011. **76**(5): p. 786-807.https://doi.org/10.1177/0003122411420817.
4. Smith, S.S., *Race and Trust.* Annual Review of Sociology, 2010. **36**(1): p. 453-475.https://doi.org/10.1146/annurev.soc.012809.102526.
5. Shelton, K.K., P.J. Frick, and J. Wootton, *Assessment of parenting practices in families of elementary school-age children.* Journal of Clinical Child Psychology, 1996. **25**(3): p. 317-329.https://doi.org/10.1207/s15374424jccp2503_8.
6. Murray, A.L., M. Eisner, D. Ribeaud, D. Kaiser, K. McKenzie, and G. Murray, *Validation of a brief self-report measure of adolescent bullying perpetration and victimization.* Assessment, 2021. **28**(1): p. 128-140,
7. Ribeaud, D., A. Murray, L. Shanahan, M.J. Shanahan, and M. Eisner, *Cohort Profile: The Zurich Project on the Social Development from Childhood to Adulthood (z-proso).* J Dev Life Course Criminol, 2022. **8**(1): p. 151-171.https://doi.org/10.1007/s40865-022-00195-x.
8. Eisner, N.L., A.L. Murray, M. Eisner, and D. Ribeaud, *A practical guide to the analysis of non-response and attrition in longitudinal research using a real data example.* International Journal of Behavioral Development, 2018. **43**(1): p. 24-34.https://doi.org/10.1177/0165025418797004.
9. Van Buuren, S. and K. Groothuis-Oudshoorn, *mice: Multivariate imputation by chained equations in R.* Journal of statistical software, 2011. **45**: p. 1-67,
10. Lüdecke, D., M. Ben-Shachar, I. Patil, P. Waggoner, and D. Makowski, *performance: An R Package for Assessment, Comparison and Testing of Statistical Models.* Journal of Open Source Software, 2021. **6**(60).https://doi.org/10.21105/joss.03139.
11. RStudio., T., *RStudio: Integrated Development for R.* RStudio, Inc., Boston, MA URL http://www.rstudio.com, 2015,
